# Discovery of holoenzyme-disrupting chemicals as substrate-selective CK2 inhibitors

**DOI:** 10.1038/s41598-019-52141-5

**Published:** 2019-11-04

**Authors:** Irina Kufareva, Benoit Bestgen, Paul Brear, Renaud Prudent, Béatrice Laudet, Virginie Moucadel, Mohamed Ettaoussi, Celine F. Sautel, Isabelle Krimm, Matthias Engel, Odile Filhol, Marc Le Borgne, Thierry Lomberget, Claude Cochet, Ruben Abagyan

**Affiliations:** 1 University of California, San Diego, Skaggs School of Pharmacy and Pharmaceutical Sciences, La Jolla, CA 92093 USA; 2 Université de Lyon, Université Claude Bernard Lyon 1, Faculté de Pharmacie - ISPB, EA 4446 Bioactive Molecules and Medicinal Chemistry, 8 avenue Rockefeller, F-69373 Lyon, cedex 8 France; 3 Pharmaceutical and Medicinal Chemistry, Saarland University, Campus C2.3, D-66123, Saarbrücken, Germany; 4 Univ. Grenoble Alpes, Inserm U1036, CEA, BCI Laboratory, IRIG, F-38000 Grenoble, France; 5 Present Address: Ecrins Therapeutics, 5 Avenue du Grand Sablon, 38700 La Tronche, France; 6 Department of Biochemistry, University of Cambridge, 80 Tennis Court Road, Cambridge, CB2 1GA UK; 7 Present Address: Cellipse MINATEC, 7 Parvis Louis Néel, 38000 Grenoble, cedex 9 France; 8 Present Address: CHU Toulouse, Emergency Department, F-31000 Toulouse, France; 9 Present Address: BioMérieux SA, Centre Christophe Mérieux, 5 rue des Berges, 38024 Grenoble, cedex 1 France; 10 Present Address: DERMADIS, 218 avenue Marie Curie, 74160 Archamps, France; 11 Centre de RMN à Très Hauts Champs, Université de Lyon, CNRS, Université Claude Bernard Lyon 1, ENS, 5 rue de la Doua, F-69100 Villeurbanne, France

**Keywords:** Molecular medicine, Targeted therapies, Kinases, Target identification, Target validation

## Abstract

CK2 is a constitutively active protein kinase overexpressed in numerous malignancies. Interaction between CK2α and CK2β subunits is essential for substrate selectivity. The CK2α/CK2β interface has been previously targeted by peptides to achieve functional effects; however, no small molecules modulators were identified due to pocket flexibility and open shape. Here we generated numerous plausible conformations of the interface using the *fumigation* modeling protocol, and virtually screened a compound library to discover compound **1** that suppressed CK2α/CK2β interaction *in vitro* and inhibited CK2 in a substrate-selective manner. Orthogonal SPR, crystallography, and NMR experiments demonstrated that **4** and **6**, improved analogs of **1**, bind to CK2α as predicted. Both inhibitors alter CK2 activity in cells through inhibition of CK2 holoenzyme formation. Treatment with **6** suppressed MDA-MB231 triple negative breast cancer cell growth and induced apoptosis. Altogether, our findings exemplify an innovative computational-experimental approach and identify novel non-peptidic inhibitors of CK2 subunit interface disclosing substrate-selective functional effects.

## Introduction

Protein-protein interactions (PPIs) play a critical role in regulation of multi-subunit protein kinases such as cAMP-dependent kinases, cyclin-dependent protein kinases and protein kinase CK2^1^, all of which have catalytic and regulatory subunits. CK2 appears as a heterotetrameric quaternary structure composed of two catalytic (α and/or α’) subunits bound to a stable dimer of two regulatory (β) subunits. In contrast to other multi-subunit protein kinases, the free catalytic α and/or α’ subunits are constitutively active, and the regulatory β subunits act as scaffolds controlling the substrate specificity and cellular localization of the holoenzyme complex^2–4^. As a pro-survival kinase, CK2 is dysregulated in various cancers and several other pathologies, thereby justifying its potential as a therapeutic target and supporting the efforts towards development of chemical inhibitors as drug candidates^5–7^. Most CK2 inhibitors identified during the last two decades are ATP-competitive molecules^8^ with one of them, CX-4945 (also known as Silmitasertib), currently investigated in clinical trials^9^ (ClinicalTrials.gov identifier: NCT02128282).

The common drawback of ATP-competitive inhibitors is their limited target selectivity, as the ATP-binding site is highly conserved among different kinases. In the field of protein kinase inhibitors, the concept of *exosites* and *exosite-targeting modulators* emerged as a way of addressing this problem. By definition, exosite targeting compounds bind outside of the ATP-site, either elsewhere on the protein kinase domain or other domains. In addition to the advantages of being non-ATP-competitive and more specific, some of these compounds possess the ability to modulate, rather than simply inhibit, the activity of the kinase, e. g. by changing its substrate preferences or subcellular localization. However, identification and validation of druggable exosites among different kinases remains challenging.

Live-cell imaging studies^10^ and the observation of an imbalance expression of CK2 subunits in various tumors^6,11^ suggested that CK2 subunits can coexist in the cell without forming the holoenzyme complex despite its remarkable stability *in vitro*^12^. The free CK2α subunit and the tetrameric holoenzyme have distinct, though overlapping, substrate specificity profiles. Thus, it could be anticipated that such a balance is a crucial point of regulation of many cellular processes governed by this multifunctional enzyme. The atomic-level understanding of the CK2α-CK2β interaction through X-ray crystallography provided the solid foundation for structure-based design of peptidic^13^ and non-peptidic (this study) small-molecule antagonists of this interaction.

The CK2α/CK2β interface is relatively small (832 Å^2^), and harbors a shallow binding pocket on the CK2α side that may be suitable for accommodating small molecules^1,14,15^ despite its high flexibility and conformational variability^16^. This exosite distinct from the catalytic cavity offers attractive opportunities for the identification of small molecules that modulate this PPI to achieve therapeutically-beneficial functional effects^15^. In search of compounds inhibiting this critical PPI, we previously designed an active cyclic peptide (Pc) derived from the CK2β carboxy-terminal domain that can efficiently antagonize the CK2 subunit interaction^13^ and a cell permeable version of this cyclic peptide (TAT-Pc) was shown to accumulate in living cells, promoting the disruption of the CK2 subunit interaction^17^. Moreover, a structural rationalization of the CK2β-competitive potential of Pc was provided by the X-ray structure of a Pc-CK2α complex^18^. Comparative molecular dynamics simulations performed on this complex highlighted, among the hydrophobic residues, the prominent role of Phe190 for a stable and active conformation of Pc^19^. Recently, improved versions of Pc were characterized with halogens in meta-position of Phe190^20^ or a novel covalent linker^21^. Notably, none of the Pc derivatives are cell-permeable, and thus they all require a cell-penetrating peptide for delivery. In parallel with efforts towards peptide-based inhibitors, a wet screening approach identified a fragment-like small molecule binding to the CK2α/CK2β interface site, CAM187^22^. Unfortunately, this compound had insufficient binding affinity and its ability to displace CK2β was not demonstrated.

Here we sought identification of better compounds candidates using structure-based virtual screening. However, similar to other kinase exosites, structure-based *de novo* discovery of CK2α/CK2β interaction inhibitors is complicated by the fact that the target site is shallow, hydrophobic, conformationally variable, and often found in poorly druggable conformations^1,23^. To overcome this hurdle, we applied a computational modeling technique to predict possible induced fit effects for small molecules and to generate pocket conformers suitable for the virtual ligand docking and screening. By virtual screening against the generated pocket conformers, we identified a lead compound that was then optimized, validated in *in vitro* assays and in cells, and crystallized to confirm the predicted binding mode. The treatment of triple-negative breast cancer cells (MBA-MB-231) with the lead candidate impeded cell growth, migration and induced cell death. Therefore, this compound is the first example of a rationally designed chemotype that efficiently displaced CK2β from CK2α in the cellular context.

## Results

### CK2 subunit interface fumigation produces druggable conformations of a target pocket

**Figure 1:**
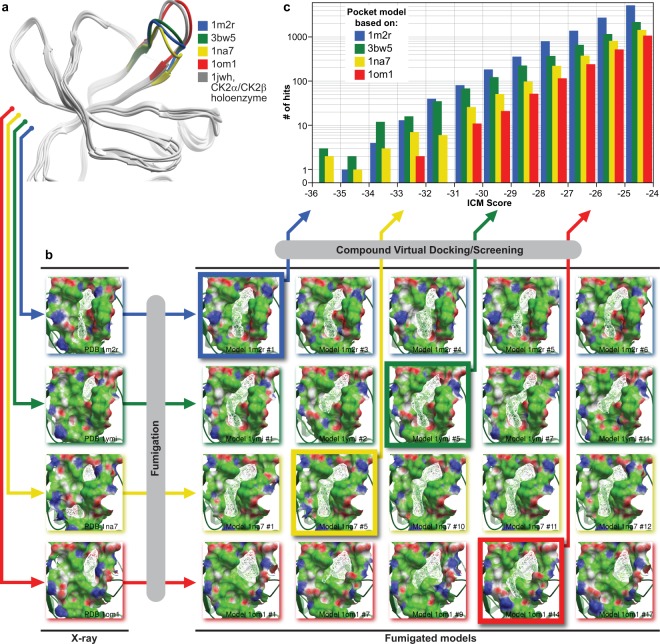
Computational identification of inhibitors of CK2α/CK2β interaction. (**a**) Ribbon diagrams of the four models of the CK2α/CK2β interface used in this study. The models differ in the position of the V101-P109 loop and demonstrate varying degree of openness of the binding site at the backbone level. The most open conformation, closely resembling the CK2β bound state of the CK2α, appears too flat to produce any appreciable small-molecule binding pockets; **(b)** The four structures of the CK2α/CK2β interface were subjected to fumigation and evaluated for druggability using ICM Pocket Finder algorithm. Fumigation resulted in larger and more drug-like pocket envelopes (white wire meshes) as compared to the original crystal structures. Four best models (framed) were selected and used for virtual ligand screening. The protein is represented by its solvent accessible surface and colored by molecular interaction properties: green – aliphatic, white – aromatic, blue – hydrogen bond (HB) donor, red – HB acceptor; **(c)** Distribution of compound binding scores predicted by ICM for the four selected fumigated CK2α/CK2β interface models. The models based on PDB 1m2r and 3bw5 (formerly 1ymi) appeared the most productive. The 1na7-based model featured the narrowest, and the 1om1-based model the widest binding pocket, both leading to the decreased number of low-scoring hits.

Binding pockets in general, and kinase *exosites* in particular, are characterized by varying degree of conformational plasticity. Molecular dynamics simulations and multiple crystal structures demonstrate the substantial plasticity of the interface regions, but rarely provide the information required for the identification of specific binding-induced sites and for determining their druggability^24^. In apo conformations, flexible elements of protein structure such as loops or side-chains often tend to *collapse* inside the pocket and obstruct the space for binding of potential ligands. The procedure of *fumigation* was designed to rearrange such collapsed apo-conformations into conformations suitable for virtual ligand screening. This approach was previously validated using three kinase exosites for which well-characterized ligands are known^25–27^. We found that the use of the fumigated models instead of the original crystallographic apo-structures improved both scoring and ranking of the active compounds in the hit list. The fumigation procedure was applied to the CK2β-binding interface of two crystal structures of human CK2α (PDB IDs 3bw5 (formerly 1ymi)^14,28^ and 1na7^29^) and two homology models built from *Zea mays* CK2α (PDB IDs 1m2r^30^ and 1om1^31^). These four models represented different degrees of openness of the binding site, controlled by the backbone positions of the loop V101-P109, in human sequence numbering, with PDB 1na7 being the most closed and PDB 1om1 being the most open (Fig. 1a). The latter structure closely resembled the CK2β-bound conformation of the loop observed in CK2α/CK2β tetramer structure (PDB 1jwh).

Following the fumigation procedure, the original interface and twenty top-scoring interface models were subjected to the ICM PocketFinder algorithm^32^ that identifies optimal ligand-binding envelopes. The model’s druggability was evaluated based on the drug-like volume and shape of the obtained envelopes. As shown in Fig. 1b, in most cases, fumigation produced pockets of larger volume and more drug-like shape than those of the original protein structure. However, it had limited success for the interface backbone conformation captured in PDB 1om1 that appeared to be too widely open to produce any appreciable ligand binding envelopes, and the envelopes in PDB 1na7 that, on the contrary, were too narrow. These models were still included in further calculations for completeness. After visual inspection, the four best models (one for each X-ray structure) were selected for virtual compound screening.

### Virtual screening against multiple fumigated pocket conformations identified potentially active compounds

A database of more than 2 million commercially available compounds was screened against the four fumigated pocket models using the ICM VLS module, with all compounds scored and rank-ordered in all four binding site models. As the ICM docking scores were optimized to distinguish strong binders from the inactive molecules, the compounds with low negative binding scores were prioritized to be tested for biological effects *in vitro* and *in vivo*. The two models based on PDB IDs 1mr2 and 3bw5 appeared to be more productive, i.e. they produced more low-scoring hits than the most open binding site structure, PDB 1om1, or the narrowest one, PDB 1na7 (Fig. 1c). The compounds with the ICM binding scores below −30 (in the ICM score units) were clustered according to their chemical structures to eliminate possible redundancy, and filtered to fit the drug-like range of such properties as molecular weight, predicted solubility and logP. The distance from each compound to the Val112 residue located at the bottom of the binding pocket was measured in order to filter out irrelevant binding poses; all compounds for which this distance exceeded 8 Å were dismissed. From the remaining compounds, 100 commercially available candidates were selected for *in vitro* evaluation.

### *In vitro* hit evaluation identified a potent inhibitor of CK2β-dependent phosphorylation

**Figure 2:**
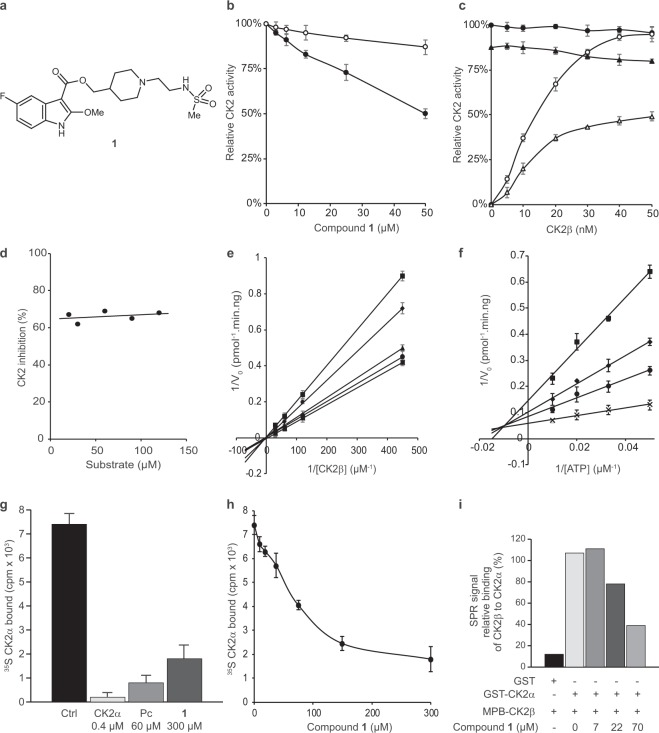
Characterization of CK2 inhibition by compound **1**. (**a**) Chemical structure of compound **1**. **(b)** Compound **1** inhibits the phosphorylation of a CK2β-dependent peptide substrate (⦁) and only shows a weak effect on the phosphorylation of a CK2β-independent substrate (⚬). **(c)** CK2α (40 nM) was incubated without (⦁,○) or with 50 μM compound **1** (▴,▵) in the presence of increasing concentrations of CK2β and assayed for phosphorylation of CK2β-independent (⦁,▴) or CK2β-dependent peptide substrates (⚬,▵). **(d)** Inhibition of CK2β-dependent phosphorylation activity by 100 μM compound **1** is non-competitive towards the CK2β-dependent peptide substrate. **(e)** Lineweaver-Burk double reciprocal plots are consistent with a competitive inhibition toward CK2β. Compound **1** concentration: 100, 50, 25, 12.5 and 0 μM. **(f)** Lineweaver-Burk double reciprocal plots are consistent with a mixed-type inhibition toward ATP. Compound **1** concentration: 100, 50, 37.5 and 0 μM. **(g**,**h)** Plate-bound MBP-CK2β was incubated with [^35^S]methionine-labeled CK2α in the absence or presence of unlabeled CK2α, Pc peptide, compound **1 (g)** or increasing concentrations of compound **1 (h)**. As a positive control, a 10-fold molar excess of untagged CK2α, was used (defined as 100% competition), and the value for 0% competition was obtained in the absence of any competitor. **(i)** GST or GST-CK2α were immobilized on biosensor surfaces and incubated in the absence or presence of the indicated concentrations of compound **1**; MBP-CK2β binding was analyzed across these surfaces using SPR technique. SPR signals are expressed as percentages of MBP-CK2β binding to GST-CK2α in the absence of compound **1**. Error bars represent the SEM of two biological replicates derived from technical triplicates.

First, all the compounds were dissolved at 200 μM in 50 mM Tris-HCl pH 7.5 buffer containing 0.1 M NaCl and 5% DMSO; the compounds that precipitated under these conditions were discarded. The 32 remaining compounds were diluted at 200 μM in the kinase buffer and centrifuged at 14,000 g. The supernatant was then assayed for ability to inhibit phosphorylation of a CK2β-dependent peptide substrate. For this, a 22-residue long N-terminal fragment of the eukaryotic translation initiation factor 2 (eIF2), known to be exclusively phosphorylated by the CK2α_2_β_2_ holoenzyme^4^ was used as a substrate. Of the 32 compounds tested, we selected compound **1** (Fig. 2a), having a molecular weight of 523.58 g/mol, that significantly inhibited the kinase in this assay.

### Compound **1** selectively inhibits phosphorylation of CK2β-dependent peptide substrate

Compound **1** inhibited phosphorylation of a CK2β-dependent peptide substrate in a dose-dependent manner (Fig. 2b), with an estimated IC_50_ of 50 μM. Under the same conditions, the phosphorylation of the canonical CK2 peptide substrate RRREDEESDDEE known to be phosphorylated equally by isolated CK2α or the holoenzyme, was only weakly affected (15% at 50 μM). Because at high concentrations, compound aggregation may sometimes lead to non-specific inhibition of kinase activity, therefore the evaluation protocol included steps designed to eliminate promiscuous aggregators^33^. Inhibition of CK2β-dependent phosphorylation by compound **1** was not affected by addition of 0.01% vol/vol Triton X-100, indicating that the inhibition is likely to be specific and not mediated by aggregation (Supplementary Fig. S1a). Additionally, the inhibition was found to be reversed by dilution of the reaction mixture or by gel filtration (Supplementary Fig. S1b,c) confirming the interaction is reversible and therefore likely to be stoichiometric binding.

### Compound **1** competes with CK2β for the binding site on CK2α

**Figure 3:**
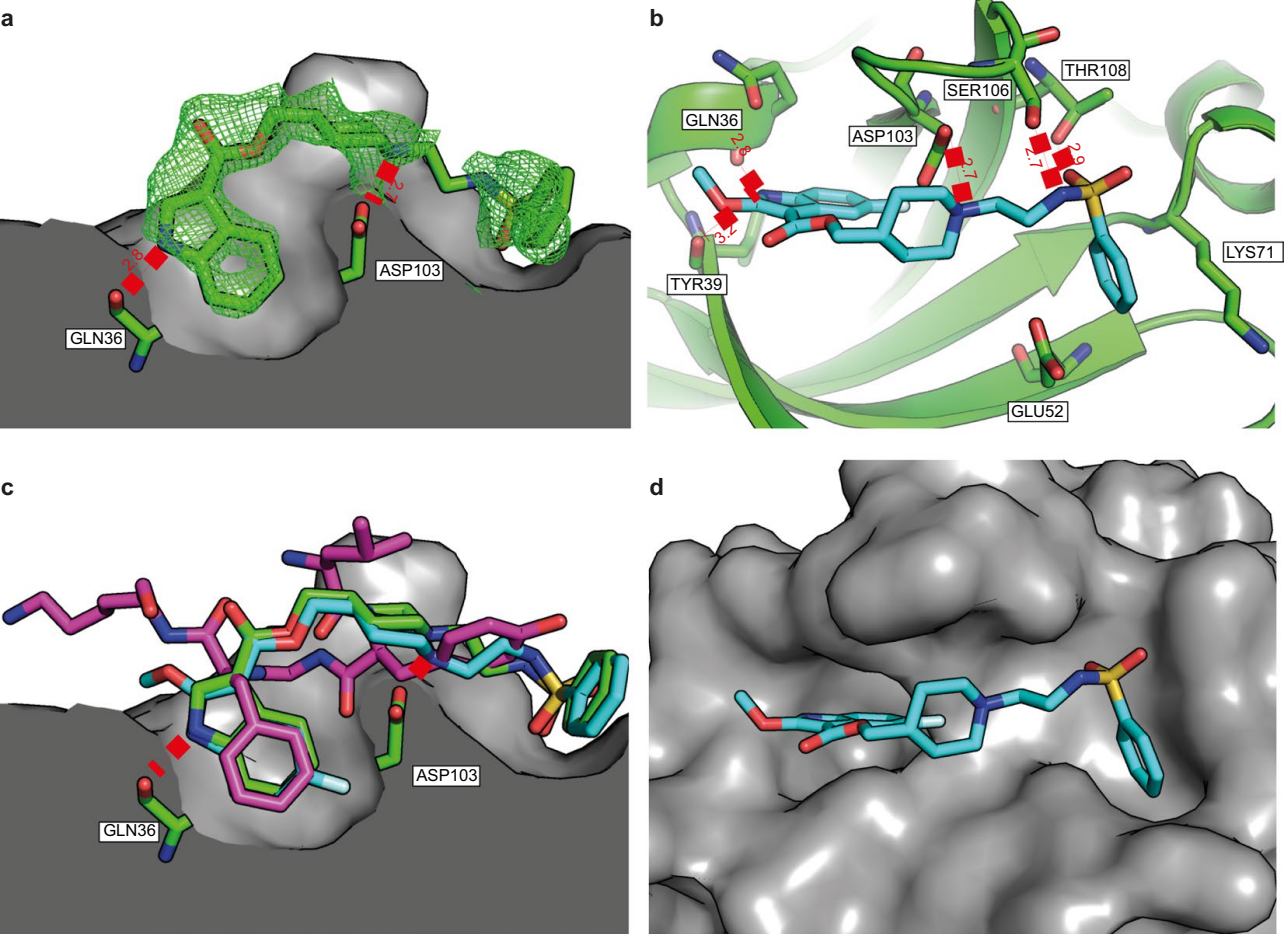
The crystal structures of **4** and **6** bound to CK2α. (**a)** The structure of **4** (green, pdb:6FVF) bound to the interface site of CK2α. CK2α is shown as the surface representation and two of the important hydrogen bond interactions with the pocket are highlighted. All distances are in Angstroms. The 2Fo-Fc map contoured at 1σ is displayed in green. **(b)** The Interactions of **6** (blue, pdb:6FVG) with the interface site of CK2α (green). All of the hydrogen bonding and salt bridge interactions are highlighted. **(c)** The superimposed structures of **4** (green), **6** (blue) and CK2β (purple, pdb:4NH1) binding in the interface site of CK2α. **(d)** The structure of **6** binding to the interface site of CK2α shown as the surface representation.

Inhibition of CK2β-dependent phosphorylation by compound **1** was antagonized by increasing CK2β concentrations (Fig. 2c), indicating that the binding of **1** and CK2β to CK2α is mutually exclusive. Steady state kinetic analysis of compound **1** complexation with CK2 was performed by incubation of CK2α with increasing concentrations of CK2β in the presence of different concentrations of the compound. ATP and eIF2-derived peptide were present at their saturating concentrations, 100 μM and 600 μM, respectively (Fig. 3e). This analysis suggests a mixed competitive inhibition towards CK2β with *K*_*i*_’ approximately equal to 65 μM for the CK2α-ATP complex.

### CK2 inhibition by compound **1** is mixed-competitive with respect to ATP and non-competitive with respect to peptide substrate

In the presence of 100 μM of compound **1**, adding increasing concentrations of CK2β-dependent peptide at a saturating concentration of ATP (100 μM) had no effect on the extent of inhibition (Fig. 2d) showing that the inhibition is also not competitive with the peptide substrate. Using steady-state kinetic analysis, we examined the effects of increasing ATP concentrations on the inhibition of phosphorylation by compound **1**. In the presence of a saturating concentration of the CK2β-*dependent* peptide substrate (133 μM), increasing ATP concentrations reduced the level of inhibition but could not restore either the maximal velocity of the enzyme, *V*_*max*_, or the Michaelis constant, *k*_*m*_, for ATP. This result suggests that compound **1** does not suppress the kinase activity in an ATP-competitive manner (Fig. 2f). *K*_*i*_ and *K*_*i*_’ value for compound **1** has been calculated from linear regression analysis of Lineweaver-Burk double reciprocal plots and found to be respectively equal to 12 μM for CK2α (Supplementary Fig. S2a) and 62 μM for the CK2α-ATP complex (Supplementary Fig. S2b).

### Compound **1** inhibits phosphorylation of a CK2β-dependent *protein* substrate

Compound **1** was further tested on a CK2β-dependent protein substrate, namely the Olig-2 transcription factor. Similar to eIF2, this 323-residue long helix-loop-helix protein is exclusively phosphorylated by the CK2α_2_β_2_ holoenzyme. Incubation of GST-Olig-2 with CK2α and increasing concentrations of CK2β in the presence of 50 μM of compound **1** led to ~50% decrease in Olig-2 phosphorylation as compared to the same experiment in the absence of inhibitor (Supplementary Fig. S3a,b). Similarly, we found that compound **1** causes a dose-dependent decrease in the autophosphorylation of CK2β by CK2 holoenzyme (Supplementary Fig. S3c–e) which was reported to depend on the formation of hetero-oligomers and supramolecular complexes^34^.

### Compound **1** directly inhibits the CK2 subunit interaction

Compound **1** was tested in an *in vitro* CK2α/CK2β interaction assay^35^. In this assay, the compound displaced soluble (^35^S) methionine-labeled CK2α from plate-bound MBP-CK2β. At a single dose concentration of 300 μM, **1** reduced the bound radioactive CK2α by about 75% (Fig. 2g). It therefore appeared to act similarly (though less potently) to the previously designed CK2β-derived cyclic peptide, Pc^13^, which inhibited ~90% of the complex formation at a concentration of 60 μM. The inhibition of the CK2α/CK2β complex formation by **1** was found to be dose-dependent (Fig. 2h). The IC_50_ (defined as the concentration of the compound necessary to inhibit 50% of the CK2α/CK2β complex formation), was found to be about 80 μM in this assay. The inhibition of CK2 subunit interaction was also studied using surface plasmon resonance (SPR). Similar to the previous experiment, the SPR signal was found to drop in the presence of increasing concentrations of **1**; at the concentration of 70 μM, **1** reduced the SPR signal by about 60% (Fig. 2i).

### Compound **1** is highly selective for CK2

Compound **1** was tested at 100 μM on a carefully selected panel of 45 serine/threonine and tyrosine kinases representative of the main human kinome families including kinases known to share similarities with CK2 such as Pim-1, HIPK, Dyrk2, CDKs, EphA4. The results presented in Supplementary Fig. S4 show that at 100 μM, compound **1** inhibited CK2α_2_β_2_ activity by 85% and CK2α activity by 27% but it had no effect on the other protein kinases apart from Pim-1, which was inhibited by 36%. Thus, among these kinases, compound **1** shows high selectivity for CK2. The Gini coefficient is commonly used to compare the selectivity of kinase inhibitors screened against panels of different kinases^36^. From the focused selectivity panel used here, the Gini coefficient of **1** was calculated to be 0.81. This is significantly higher than the clinical trial candidate CX-4945 which has a Gini coefficient of 0.62 and comparable to the most selective CK2α inhibitor reported to date, CAM4066, which has a Gini coefficient of 0.82^37^. These results highlight the potential of targeting kinase exosites as a promising strategy for the development of highly selective inhibitors.

### Optimization of compound **1** based on the predicted binding pose at the interface

The predicted binding pose for compound **1** is shown in Supplementary Fig. S5. The fluorinated indole ring is buried in the hydrophobic part of the binding pocket formed by Y39, L41, F54, A56, V67, V101, P104, V105, A110, and V112. In addition, the polar NH of the indole ring and the methoxy ether oxygen are likely to interact with the backbone amides of Q36, D38, and Y39. *Via* a polar ester linker, the ligand extends towards the top of the kinase P-loop, where it makes multiple polar and hydrophobic interactions. Compound **1** contains no toxicophores or reactive groups and as such is a valuable hit for validating the concept of disrupting the CK2 subunit association as a selective way to inhibit the kinase activity. However, its limited potency prevented further elucidation of its cellular effects. Therefore, compound **1** was used as a starting point for further development and structure-activity relationship (SAR) studies, guided by the predicted binding pose. Different modifications of **1** were envisaged such as: (1) the removal of the fluorine atom and the methoxy group at positions 5 and 2 of the indole ring, respectively and (2) the replacement of the methylsulfonamide group by a phenylsulfonamide. The synthetic strategy for the preparation of analogues **2**–**6** of the hit compound **1** was inspired by a literature procedure used for 5HT_4_ receptor antagonists^38^ and is reported in the Supplementary Information.

Compounds **2**–**6** were first evaluated for their ability to inhibit the phosphorylation of CK2β-dependent CK2 activity (Table 1). Analogues **2** and **3** were less active than the parent compound **1**, indicating a crucial role for methoxy and fluorine substituents in this methylsulfonamide series. Moreover, a significant improvement in the inhibition of CK2β-dependent peptide phosphorylation was noticed after the introduction of a phenylsulfonamide group at the extremity of the side chain, as noticed by the increased inhibitory potency of compounds **4**–**6**. The most active compound **6** and its unsubstituted analogue **4** were chosen to complete their biophysical and cellular evaluations, in order to confirm a protein-protein interaction inhibition mode. When tested in enzymatic assays, **6** and **4** showed the highest inhibitory activity toward the phosphorylation of CK2β-dependent peptide substrate with IC_50_ of 22 and 45 μM, respectively (Table 1 and Supplementary Fig. S6a). Like compound **1** (Fig. 2c), compound **6** strongly inhibited the phosphorylation of the CK2β-dependent peptide substrate, while the phosphorylation of the CK2β-independent peptide substrate was only weakly affected (Supplementary Fig. S6b).

**Table 1:** Optimization of compound 1.

Compound	Structures	Inhibition % at 50 μM	IC_50_ (μM)	*K*_*D*_ (μM)
**1**		48	50	Nd
**2**		25	nd	44 ± 6
**3**		28	nd	54 ± 5
**4**		57	45	41 ± 5
**5**		66	nd	43 ± 2
**6**		80	22	30 ± 2

*In vitro* CK2 inhibition in the presence of compounds **1**–**6** at 50 μM was assayed with a CK2β-dependent peptide substrate as described in Experimental Procedures (SEM of two biological replicates derived from technical triplicates <20%). The *K*_*D*_ values were determined by surface plasmon resonance (nd: not determined).

### Determination of binding kinetics using surface plasmon resonance (SPR)

In order to determine the affinity constants and the binding kinetics of our small set of molecules, we performed SPR analysis after coupling GST-CK2α to the biochip surface. Intriguingly, the strengthened inhibition observed in the enzymatic assay upon addition of a phenyl on the sulfonamide moiety was not fully correlated with the affinity (*K*_D_) for CK2α as measured in the SPR experiments. However, the *K*_a_ and *K*_d_ derived from the SPR analysis revealed that this modification significantly decreased the off-rate, for example, from 19 ± 2.10^−2^ s^−1^ to 5.2 ± 0.1.10^−2^ s^−1^ for compounds **2** and **5**, respectively (Supplementary Fig. S7). The same effect was observed for the other pairs of compounds differing only by this phenyl moiety. Notably, this reduced off-rate has been shown to be favorable for the target residence time and hence the biological activity^39^.

### Investigation of the binding mode and site using NMR experiments

NMR experiments were performed for the compound exhibiting the highest solubility in the protein buffer, i. e. compound **4**. The STD-NMR spectrum^40^ recorded for compound **4** bound to CK2α reflects its binding mode through the discrimination of the solvent-exposed protons from the buried protons of the inhibitor bound to its receptor. As illustrated in Supplementary Fig. S8a,b, the indole moiety is the most buried region of the inhibitor bound to CK2α. The orientation of the indole moiety inferred from the STD analysis is also in full agreement both with the predicted mode shown in Supplementary Fig. S5. In addition, STD-NMR was used to analyze the competition between compound **4** and AMPPNP, a non-hydrolysable ATP analogue. As shown in Supplementary Fig. S8c, AMPPNP is not displaced upon compound **4** binding, thus confirming that this inhibitor does not bind in the ATP binding site.

### Crystal structure of CK2α-**4** and CK2α-**6** complexes

Crystal structures were determined of both **4** and **6** bound to the CK2β binding site on CK2α (pdb: 6FVF and 6FVG). To accommodate the compounds, the β4-β5 loop moves out to open up the pocket compared to the apo structure (pdb: 5CU6)^37^. As predicted by modeling and suggested by the STD analysis, the hydrophobic ring of the indole from **6** and **4** bind in the pocket occupied by Phe190 of the CK2α:CK2β complex (Fig. 3a–c). Indeed, the crystal structures of **4** and **6** show a very good alignment of the indole and the phenyl ring from the CK2 complex (pdb:4DGL)^23^. The additional fluorine in the 4-position of **6** more effectively fills the side of the pocket (Fig. 3c). The nitrogen of the indole interacts with the polar residues of the backbone amides from Gln36 and Tyr39 (Fig. 3a,b) as predicted by the modelling. Interestingly, the OMe group of **6** does not appear to be making any additional interactions with the binding site (Fig. 3b). However, it may affect binding due to its electron donating properties. The nitrogen from the piperidine ring in both compounds is positioned to make a salt bridge interaction with the carbonyl of Asp103 (Fig. 3a,b). The electron density for the linker between the piperidine and the phenylsulfonamide is poor suggesting that the linker is partially disordered and not making any favorable interactions with the protein (Fig. 3a). However, there is definite electron density for the sulfonamide at the end of the short linker. The sulfonamide can clearly be seen making hydrogen bonding interactions with Ser106 and Thr108 of the β4-β5 loop (Fig. 3b). The phenyl group then sits in the shallow pocket created by Lys71, Glu52 and Arg47 (Fig. 3d).

### Compound **6** disrupts the interaction of CK2 subunits in cells

**Figure 4:**
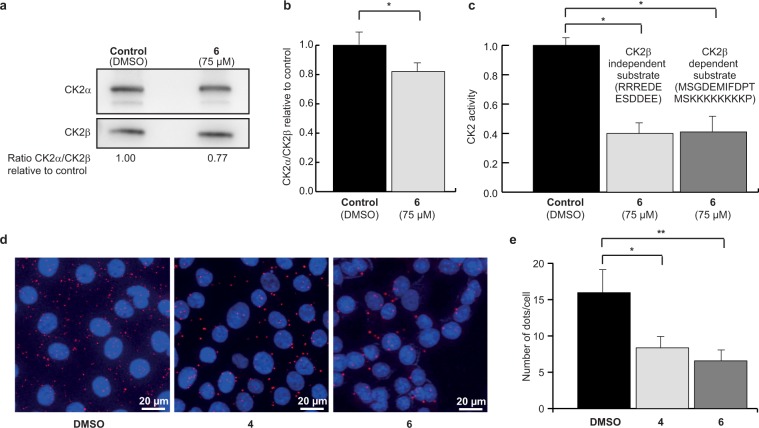
Uptake and cellular effects of compound 6. Anti-CK2β immunoprecipitates were prepared from MCF10A cells incubated with DMSO (0.5%) or 75 μM **6** for 4 h. The corresponding immunoprecipitates were analyzed for the presence of CK2 subunits by Western blot **(a)** and quantified in **(b)** or for CK2 activity with CK2β-independent and CK2β-dependent peptide substrate **(c)**. Data are representative of three biological replicates, uncropped blots are shown in Supplementary Fig. S16 and statistical analysis using Wilcoxon signed rank test showed a p-value of 0.03 in both cases (*). **(d)** *In situ* proximity ligation images of MCF10A cells incubated with 50 μM **6** for 3 h. **(e)** Number of fluorescent dots per cell was quantified using the BlobFinder software. n = 120 cells from two independent experiments. Mann Whitney test was used and results were found significant (*p-value = 0.0003 and **p-value = 0.044).

The transient nature of the CK2 holoenzyme has been highlighted by the elucidation of its structure^1^. Furthermore, analysis of the spatiotemporal organization of individual CK2 subunits in living cells has shown that they are highly dynamic^10,12^. Thus, we evaluated the effect of **6** on the CK2 subunit association in human mammary epithelial cells, using two independent strategies. First, CK2β was immunoprecipitated from extracts of MCF10A cells treated with DMSO as control or 75 μM of **6**, and the amount of CK2α or CK2 catalytic activity recovered in the immunoprecipitates were analyzed by western blot and CK2 activity assay. Significantly less CK2α co-immunoprecipitated with CK2β when cells were incubated with **6** (Fig. 4a,b), compared to the DMSO-treated control. The measured CK2 activity was also significantly lower in precipitates from cells treated with **6** (Fig. 4c), both towards a CK2β-dependent and a CK2β-independent substrate (MSGDEMIFDPTMSKKKKKKKKP and RRREDEESDDEE, respectively, see Methods). These findings suggest that **6** inhibited the high affinity interaction of the CK2 subunits in living cells.

To confirm these results, the effect of **6** and **4** was investigated using the *in situ* proximity ligation assay (PLA), a method of choice to evaluate a protein-protein interaction in living cells as it enables the direct observation of protein interactions in unmodified cells^41^. Significantly fewer CK2 holoenzyme complexes were observed in MCF10A cells treated with 50 μM of **6** (6 ± 2 dots/cell) and **4** (8 ± 2 dots/cell) compared to DMSO control (16 ± 4 dots/cell) (Fig. 4d,e). Most of the complexes were found in the cytoplasm, although some complexes were observed in the nucleus as well, and the relative distribution of complexes between the two compartments appeared unchanged upon cell treatment with **4** or **6**. This is consistent with the notion of the compounds equally diffusing into all cell compartments, which is typical for small drug-like compounds, and presents a potential advantage over the previously described vectorized peptides^20,21^.

Collectively, these results demonstrate that both **6** and **4** disrupt the dynamic CK2 subunit interaction in living cells.

### Compound **6** inhibits cell growth, migration and triggers cell death

**Figure 5:**
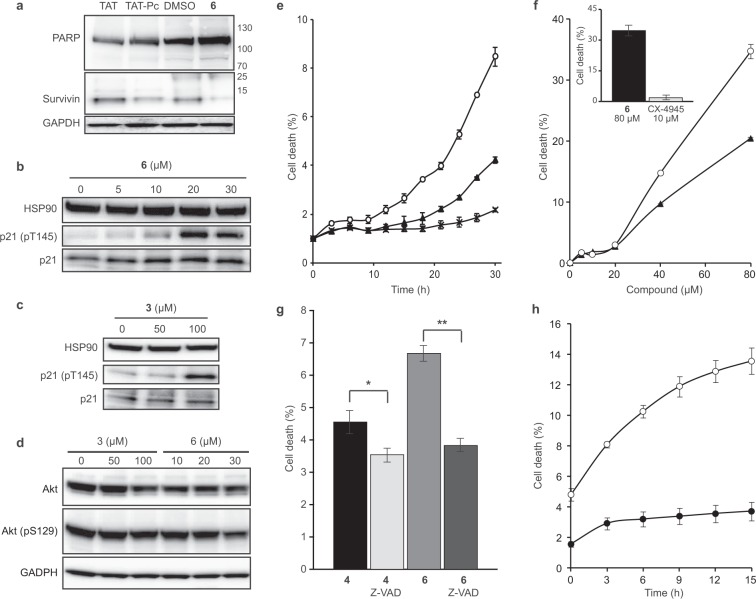
Cellular effects of compounds **3**, **4** and **6**. MCF10A cells were incubated for 24 h with either DMSO or 30 μM TAT or 30 μM TAT-Pc or 25 μM **6** (**a**) or with increasing concentrations of **6 (b)** or **3 (c)**. MCF10A cells were incubated for 48 h with either DMSO or with increasing concentrations of **3** or **6 (d)**. Cells were then lysed and analyzed by western blot with the indicated antibodies. Blot images are representative of at least three independent experiments. Uncropped blots are shown in Supplementary Fig. S16. **(e)** MDA-MB231 cells were treated with 40 μM **6** (⚬), **4** (▴) or **3** (**X**). **(f)** MDA-MB231 cells were treated for 30 h with increasing concentrations of **6** (⚬) or **4** (▴). Insert: cells were treated for 30 h with 80 μM **6** or 10 μM CX-4945. **(g)** Cells were incubated with 80 μM **6** or **4** in the absence or presence of 20 μM Z-VAD. Statistical analysis using a two-way ANOVA with multiple comparison, Uncorrected Fisher’s LSD, showed that cell death was not significantly affected by Z-VAD for **4** (p = 0.1907, *) but was significantly affected for **6** (p = 0.0006, **). **(h)** MDA-MB231 cells (⚬) or MCF10A cells (⦁) were incubated for 24 h with 80 μM **6**. Cell death was automatically quantified from images captured every 3 h for the duration of the experiments using an Essen IncuCyte Zoom live-cell microscopy incubator. Error bars represent the SEM of two biological replicates derived from technical triplicates.

In order to investigate the intracellular events induced by **6**, MCF10A cells were incubated with either the cell permeable CK2β-derived cyclic peptide inhibitor of CK2α/β subunit interaction (TAT-Pc), or compound **6**, after which cell extracts were analyzed by western blot. TAT-Pc was previously described as an inducer of cell death^17^. Caspase-dependent PARP cleavage is an early event of apoptosis, the process of programmed cell death^42^. The presence of an 89 kDa PARP fragment is routinely used as a reporter of apoptosis. No significant PARP cleavage was detected in cells treated with **6** or TAT-Pc. However, both **6** and TAT-Pc decreased survivin expression, a well-known inhibitor of caspase-9 and apoptosis blocker^43^ (Fig. 5a). It has been previously shown that in response to various stresses, the expression of the cell cycle inhibitor p21 is upregulated, leading to cell growth arrest. Activated Akt phosphorylates p21 at Thr145 promoting its cytoplasmic retention in breast cancer cells^44,45^. Previously, p21 phosphorylation was used as an indirect reporter of CK2 activity^46,47^ and a downregulation of this specific phosphorylation was reported in response to the ATP-competitive CK2 inhibitor CX-4945^46^. By contrast, TAT-Pc triggers phosphorylation of p21 at Thr145^17^. Similarly, we observed a striking dose-dependent increase of p21 Thr145 phosphorylation in response to cell treatment with compound **6**, reaching the maximum at a concentration of 20 μM (Fig. 5b). Of note, the structurally closely related but less potent analog **3** showed a moderate increase of p21 phosphorylation at a concentration of 100 μM (Fig. 5c), thus corroborating the SAR in the cellular context. Another frequently used reporter of CK2 activity in a cellular context is Akt phosphorylation at Ser129. Accordingly, a slight but not statistically significant alteration of Akt phosphorylation at Ser129 was detected in cells treated with 100 µM of **3** or 30 µM of **6** (Fig. 5d).

To further explore the kinetics of cell death, we tracked MCF10A or MDA-MB231 cells by live cell imaging for extended periods of time following exposure to **6**, **4** or **3**. The percentage of cells undergoing cell growth inhibition or cell death was automatically quantified using the IncuCyte ZOOM™ live-cell imaging and analysis system (Essen BioScience, Inc.). The efficacy of **6** was first tested on the cell growth inhibition of MCF10A cells (Supplementary Fig. S9a). This assay yielded a GI_50_ of 22 ± 3 μM for **6**, in full accordance with the observed increase of p21 phosphorylation and thus indicating that no significant change in its potency was observed from biochemical to cellular assays (see Fig. 2b). We then compared the efficacy of **6** on WT MCF10A or CK2β-depleted MCF10A cells (ΔCK2β) which harbors 80% less CK2β than their WT counterpart. The experiment depicted in Supplementary Fig. S9b showed that the growth inhibition induced by **6** was twice more pronounced in CK2β-depleted cells than in WT cells, thus corroborating that this chemical inhibitor behaves as a CK2β antagonist in living cells. This is consistent with the observation that compound **1**-mediated CK2 inhibition *in vitro* can be attenuated by increasing CK2β concentrations (Fig. 2c).

The effect of **6** on the *cell migration* of MCF10A and MDA-MB231 cells was assessed in a scratch wound assay using the Essen Bioscience WoundMaker^TM^. In this assay, untreated MCF10A and MDA-MB231 cells closed the wound at 20 h and 48 h respectively (Supplementary Fig. S10a,b). Migration of both cell lines was completely abrogated by **6** whereas wound closure was not affected by the inactive analog **3**. This analysis also revealed that following **6** or **4** treatment, the majority of MDA-MB231 cells rapidly retracted membrane extension (in 9 h), followed (an average of 3 h later) by the appearance of dead cells (Fig. 5e and Supplementary Fig. S11a,b). The structurally related but inactive analog **3** was without effect in this assay (Fig. 5e). As shown in Fig. 5f, both **6** and **4** caused cell death that was already detectable at a concentration of 20 μM, and became massive at 80 μM. Compared to CX-4945, **6** was much more efficient in inducing cell death (Fig. 5f inset). As expected, **4** was also efficient to compromise the viability of MDA-MB231 cells (Supplementary Fig. S11a).

Finally, we evaluated the effects of compounds on apoptosis. A hallmark of apoptosis is externalization of phosphatidylserine on the cell membrane prior to loss of cell membrane integrity, which can be monitored by annexin V/propidium iodide staining. Time-course analysis of apoptosis by AV/PI staining of MDA-MB231 cells treated with 40 μM of **6** revealed that apoptosis was detected after only 8 h of exposure. Importantly, under the same experimental conditions, the less potent analog **3** was unable to induce apoptosis (Supplementary Fig. S12a,b). Consistent with caspase-dependent apoptosis, the pan-caspase inhibitor, z-VAD-FMK, significantly attenuated the loss in cell viability induced by **6** (Fig. 5g). Interestingly, we found that Triple-Negative MDA-MB231 cells were much more sensitive to **6**-mediated cell death than MCF10A cells (Fig. 5h).

## Discussion

Exosite-targeting inhibitors and modulators of protein kinase activity have attracted increasing attention in recent years, due to their potential for high kinase specificity and, most importantly, the opportunity to modulate the kinase activity in a specific manner. The *de novo* structure-based discovery of such compounds, however, presents a substantial challenge, as their target sites are often shallow and conformationally variable, thus preventing the direct use of the pocket conformations found in crystallographic structures^48^.

In this work, we presented a modeling protocol called *interface fumigation* that can be applied to kinase exosites and other challenging apo sites in order to generate more druggable pocket conformations suitable for virtual ligand docking. The alternative pocket conformations are generated by conformational sampling in the presence of a repulsive density representing a generic yet unknown ligand. While such procedure inevitably leads to an increase in conformational strain of the pocket side-chains, it is well known that the ligand-bound state of the protein rarely or never represents the minimum of its Gibbs free energy; it is the energy of the complex that is optimized.

Application of the presented fumigation protocol to the CK2β-binding interface on CK2α followed by druggable pocket selection and virtual screening led to identification of compound **1**, a potent inhibitor of CK2α/CK2β interaction. This compound prevented the formation of the holoenzyme complex and inhibited phosphorylation of CK2β-dependent substrates. Enzymatic and biophysical characterization confirmed that the inhibition was achieved *via* binding to the CK2α/CK2β interface. Compound **1** suppressed the subunit association *in vitro*. The inhibition was found to be mixed-competitive towards ATP, non-competitive towards the peptide substrate, and was reversed by the addition of CK2β. However, the limited potency of compound **1** prevented further elucidation of its cellular effects. Therefore, compound **1** was used as a platform for structure-based rational design of more potent analogs. A series of five strategically designed analogs was synthesized. Among them, **6** and **4** showed improved CK2 inhibitory activity but, more importantly, demonstrated suitable properties to disrupt the CK2 subunit interaction in living cells.

The crystal structures of **4** and **6** clearly show that both compounds bind at the CK2α/CK2β interface and block the interaction between the two, which accounts for the specific inhibition of CK2β dependent substrates. However, in addition to affecting the interface site, we observed an allosteric effect: the binding of **4** and **6** also causes the transition of the αD loop from the open to the closed conformation^49^ (supplementary Fig. S13a). Interestingly, the electron density for ATP in the structures is very weak compared to that observed in the apo form of the crystal structure. This implies that ATP is being partially destabilized by the binding of **4** or **6** to the CK2α/CK2β interface site. Analysis of the crystal structure shows that the β4-β5 loop is pushed open to allow the binding of **4** or **6** (supplementary Fig. S13b). The β4-β5 loop is closely linked to the hinge region (supplementary Fig. S13b) and its movement may therefore cause the shift in the hinge region observed in both the crystals structures. The hinge region interacts directly with the adenosine moiety of ATP in the apo structure (pdb:5CU6 supplementary Fig. S13c), and its movement therefore disrupts the hydrogen bonding network with ATP. Displacement of hinge binding compounds (e.g. ATP) has been observed in association with a unique closed conformation of the αD loop^37^. Therefore, this is a likely mechanism by which the αD loop is forced into the closed position. However, it may also be that the movement of the β4-β5 loop directly causes the αD loop to adopt the closed form which in turn leads to the displacement of ATP. Whichever mechanism is in operation, the resulting displacement of ATP may account for the mixed-competitive mode of inhibition with respect to ATP and the lower K_*i*_ for CK2α than for the CK2α-ATP complex that was observed for these compounds.

One of the key advantages in targeting kinase exosites (as opposed to the ATP-site) is the ability to achieve highly selective inhibition. Accordingly, compound **1** was selective for CK2 when tested against a panel of 45 human protein kinases representing all families, with the only two exceptions being a mild effect on Pim-1 and CHK2 (36% and 24% inhibition at 100 μM, respectively). The degree of conservation of the compound binding interface between CK2α and these two kinases is low (Pim-1: 7.7% identity and 25.3% similarity to CK2α; CHK2: 25% identity and 36.8% similarity to CK2α; numbers calculated over the 15 residues making side-chain contacts with the compound in the predicted binding pose); therefore, it is unlikely that the compound inhibits Pim-1 thorough a similar mechanism.

An interesting and yet unanswered question is whether the compounds reported here interfere with CK2β binding to other kinases known to be subject to its regulatory activity: A-Raf^50,51^, Mos^52,53^, and CHK1^54^. These kinases were not part of the 45-kinase selectivity panel. Moreover, with the exception of the CK2α_2_β_2_ holoenzyme, the panel was focused on monomeric kinases; therefore, even if these kinases were included, we would likely have missed compound effects on their interaction with CK2β or phosphorylation of CK2β-dependent substrates. As it stands, the investigation of the inhibitory activities of the identified compounds on CK2β regulation of kinases other than CK2α remains subject to future exploration.

### Significance

Despite CK2 being in the focus of biomedical research for more than 50 years, the mechanisms of its regulation and its role in health and disease are still not completely understood. CK2 is recognized as a systemic pro-survival enzyme which under certain conditions may contribute to cancer development, and as a potential target in several neoplastic diseases. The activity, substrate specificity and subcellular localization of the CK2 holoenzyme is determined by its CK2β subunits. Therefore, the interaction between the CK2 subunits has central importance and it could be predicted that such a balance is crucial to control the numerous cellular processes that are governed by this multifaceted enzyme^12^. Blocking this interaction could represent an unconventional way to partially inhibit CK2 activity, specifically antagonizing the CK2-holoenzyme-mediated phosphorylation of CK2β-dependent protein substrates, providing a less toxic approach to target these substrates rather than total CK2 enzymatic inhibition.

The substrate-selective CK2 inhibitors identified in this study can further help understanding the sophisticated mechanisms of regulation of intracellular CK2. They may be used as research tools for identification of CK2β-dependent substrates using phospho-proteomic approaches, or as a basis for the structure-based design of a next generation of CK2 inhibitors. The selective disruption of the CK2α/CK2β interaction also provides possibilities to pharmacologically test the importance of this interaction in tumor cell growth and viral infections^55^.

## Methods

### Evaluation of model druggability with ICM PocketFinder

Interface druggability was evaluated by the ICM PocketFinder algorithm^32^ as implemented in ICM version 3.5. The algorithm starts by calculation of the Lennard-Jones potential around the protein a 3D grid with 1 Å step size. The grid map value at point **r** is given by:

1 $${P}_{0}({\bf{r}})=\sum _{a}[\frac{{A}_{a}}{{d}^{12}(a,{\bf{r}})}-\frac{{B}_{a}}{{d}^{6}(a,{\bf{r}})}]$$

where the sum is taken over all atoms *a* in the system, *d*(*a*, **r**) is the distance from atom *a* to the grid point **r**, and the atom-dependent parameters *A*_*a*_ and *B*_*a*_ are taken from the Empirical Conformational Energy Program for Peptides (ECEPP)/3 molecular mechanics force field. Map values *P*_0_(**r**) are truncated at −0.8 kcal/mol to retain only the attractive regions.

Next, Gaussian convolution of the potential *P*_0_ at each grid point **r** is performed by:

2 $$P({\bf{r}})=\frac{1}{{\lambda }^{3}}\int \exp (\,-\,\frac{{d}^{2}({\boldsymbol{x}},\,{\boldsymbol{r}})}{{\lambda }^{2}}){P}_{0}({\bf{x}})d{\bf{x}},\,\lambda =2.6\AA $$

where *d*(**x**, **r**) is the distance between grid points **x** and **r**. The resulting map *P*(**r**) is contoured using an in-house algorithm to produce envelopes whose location, shape and volume were indicative of the ligand binding pockets.

### Interface pocket model generation by fumigation

Two X-ray structures of human CK2α (PDB IDs 3bw5^28,56^ and 1na7^29^) and two homology models built from *Zea mays* (PDB IDs 1m2r^30^ and 1om1^31^) were used as the starting points for pocket simulations. For each model, its largest possible pocket volume was approximated by a space density calculated as follows:

1. Simultaneous conversion of pocket side-chains (except Ala, Gly, and Cys) to Ala,
2. Construction of atom density map for the obtained “shaved” protein on a 0.5 Å 3D grid by
  3 $$D({\bf{r}})=\sum _{a}exp(-\frac{{d}^{2}(a,{\bf{r}})}{{w}_{a}^{2}})$$
  Here *w*_*a*_ is the Van der Waals radius of atom *a*.
3. Gaussian convolution of the potential according to Eq. (2) with *λ* = 12 Å to obtain a less detailed map *D*_c_(**r**), filling the cavities of the original protein.
4. Taking a difference of the convoluted and the original maps, *F*(**r**) = *D*_c_(**r**) − *D*(**r**)

The side-chains of all four interfaces were sampled using ICM biased probability Monte Carlo sampling procedure in internal coordinates^57,58^, with the generated fumigation map *F*(**r**) included as a penalty term in the combined energy function (Supplementary Fig. S14a–c).

### ICM grid docking

ICM molecular modeling software^57,58^ version 3.5 was used for ligand docking and scoring. ICM ligand docking is based on biased probability Monte Carlo optimization of the ligand internal coordinates in the set of grid potential maps of the receptor^59^. Compounds in two-dimensional representation were converted to 3D and optimized using MMFF-94 force field. The generated conformers were then placed into the binding pocket in four principal orientations and used as starting points for Monte Carlo optimization. The optimized energy function included the ligand internal strain and a weighted sum of the grid map values in ligand atom centers.

### ICM full-atom scoring

The top-scoring ligand poses were merged with their receptors to obtain full-atom models of the complexes which were evaluated with full-atom ICM ligand binding score^60,61^ that has been previously derived from a multi-receptor screening benchmark as a compromise between approximated Gibbs free energy of binding and numerical errors. The score was calculated by:

4 $${S}_{bind}={E}_{int}+T\Delta {S}_{Tor}+{E}_{vw}+{\alpha }_{1}\times {E}_{el}+{\alpha }_{2}\times {E}_{hb}+{\alpha }_{3}\times {E}_{hp}+{\alpha }_{4}\times {E}_{sf}$$

where *E*_*vw*_, *E*_*el*_, *E*_*hb*_, *E*_*hp*_, and *E*_*sf*_ are Van der Waals, electrostatic, hydrogen bonding, non-polar and polar atom solvation energy differences between bound and unbound states, *E*_*int*_ is the ligand internal strain, Δ*S*_*Tor*_ is its conformational entropy loss upon binding, T = 300 K, and *α*_*i*_ are ligand- and receptor-independent constants.

### Virtual ligand screening

From the pocket conformation ensembles generated by the fumigation algorithm, four interface models were selected for Virtual Ligand Screening. Each model was converted into a set of potential grid maps, and a database of 2,285,435 commercially available compounds (Molcart, Molsoft LLC) was screened against them using standard ICM virtual screening protocol as implemented in ICM version 3.5. Top 100 compounds were selected for experimental validation.

### Compounds

All the 100 chemical compounds that were selected after the virtual screening for the *in vitro* evaluation were reagent grade or better and were supplied by the following companies: Chembridge (San Diego, CA), ChemDiv (San Diego, CA), Enamine (Kiev, Ukraine), InterBioScreen (Moscow, Russia), LifeChemicals (Kiev, Ukraine), Maybridge/Fisher Scientific (UK), National Cancer Institute, Pharmeks (Moscow, Russia), Sigma-Aldrich. Compounds were dissolved at 10 mM in DMSO and stored at −20 °C.

### Synthesis of compounds **2**–**6**

The synthetic route and chemistry procedures to synthesize key intermediates and compounds **2–6** are fully described in the Supporting Information (Fig. S15 and Supplementary Materials and Methods). Additional references are also given.

### *In vitro* CK2α/CK2β interaction assay

The CK2α/CK2β interaction assay involved competition between plate-bound MBP-CK2β and the selected compounds for binding to soluble [^35^S]methionine-labeled CK2α (10^5^ cpm). The assays were performed in reacti-Bind streptavidin-coated high-binding-capacity 96-well plates (Pierce) in which each well was coated with 250 ng of biotinylated MBP-CK2β in 50 mM Tris/HCl, pH 7.2, and 0.4 M NaCl buffer. The compounds and [^35^S]methionine-labeled CK2α were added in each well in the same buffer, incubated for 1 h at room temperature and triple-washed, after which the radioactivity of each well was determined using a scintillation counter. Positive control (100% competition) was determined with a 10-fold molar excess of untagged CK2α, and negative control (0% competition) was performed in the absence of competitor^35^.

### CK2β-independent and CK2β-dependent peptide substrates

The following peptide substrates were employed: a canonical CK2 peptide substrate RRREDEESDDEE phosphorylated equally by CK2α and CK2α_2_β_2_ (*CK2*β*-independent peptide substrate*), and MSGDEMIFDPTMSKKKKKKKKP exclusively phosphorylated by CK2α_2_β_2_ (*CK2β-dependent peptide substrate)*^4^. GST-Olig2-(1-177), was used as a *CK2*β*-dependent protein substrate*^13^.

CK2 kinase assays were performed in a final assay volume of 18 μL containing 3 μL of compounds, 3 μL of CK2α (36 ng) and a mixture of 1 mM peptide substrate, 10 mM MgCl_2_, and 1 μCi [γ^32^P]-ATP. Final concentration of ATP was 100 μM. Assays were performed under linear kinetic conditions for 5 min at room temperature before termination by the addition of 60 μL of 4% TCA. ^32^P incorporation in peptide substrate was determined as previously described^62^.

For Olig2 phosphorylation assay, GST-Olig2 fusion protein (3.7 μg) was incubated with 200 nM CK2α, in the absence or presence of 50 μM compound **1** and increasing concentrations of CK2β. Samples were analyzed by SDS PAGE and subjected to autoradiography. Phosphoproteins were quantified by densitometry scanning. For eIF2 peptide phosphorylation assay, the CK2β-dependent peptide substrate (600 μM) was incubated with CK2α (40–50 nM) and increasing concentrations of compound **1** or/and CK2β.

### Construction of plasmids and protein expression

Human recombinant CK2α subunit was expressed in *Escherichia coli* and purified to homogeneity as previously described^63^. Expression and purification of chicken recombinant MBP (maltose-binding protein)-CK2β were performed as described previously^64–66^. Proteins were quantified by a Bradford assay, and the quality of the purification was asserted by SDS-PAGE analysis.

### Kinase selectivity profiling

Kinase selectivity of compound **1** was assessed using a panel of 45 recombinant protein kinases. The assays were performed at 10 μM ATP in the presence of 100 μM inhibitor using the Kinase profiler panel service (Millipore). Inhibition, expressed as the percent of activity determined in the absence of inhibitor, was calculated from the residual activity measured in the presence of 100 μM inhibitor.

### Surface plasmon resonance

SPR competition studies (Fig. 2i) were performed using a BIAcore 3000 instrument (BIAcore AB) equipped with a CM5 sensor chip (BIAcore AB). After activation with a solution of N-hydroxysuccinimide and N-ethyl-N-(dimethylaminopropyl)carbodiimide hydrochloride (coupling solution BIAcore AB), the anti-GST antibody diluted at 30 μg/ml in 10 mM acetate buffer pH 5.0 was injected at a flow rate of 5 μL/min for 7 min. All the surfaces were blocked with a 3 min injection at 10 μL/min of 1 M ethanolamine, pH 8.0. GST-CK2α (50 μg/ml) was immobilized on the surface at a flow rate of 5 μL/min in HBS (10 mM Hepes pH 7.3, 0.15 M NaCl, 3 mM EDTA, 0.005% polysorbate 20). MBP-CK2β diluted at 50 μg/ml in HBS buffer was injected over the surface at a flow rate of 5 μL/min in presence of various compound **1** concentration. Regeneration of the surfaces was achieved by injection of 10 mM glycine-HCl pH 2.2. SPR binding studies (Supplementary Fig. S7) were performed using a Reichert SR7000DC instrument optical biosensor (Reichert Technologies) equipped with a CMD500m sensor chip obtained from XanTec Bioanalytics. CK2α was immobilized by a capturing approach using a monoclonal anti-GST antibody (27 kDa, Clone GST-R 6G9 produced in rat, SAB4200055 Sigma Aldrich). Prior to use, the anti-GST antibodies were purified twice by micro dialysis at 4 °C (Membrane: dialysis tubing benzoylated, 9 mm, D2272-5FT) in 10 mM sodium acetate, pH = 5. Anti-GST antibodies were immobilized using amine-coupling chemistry at 12 °C. The surfaces of all two flow cells were activated for 7 min with a 1:1 mixture of 0.1 M NHS (N-hydroxysuccinimide) and 0.1 M EDC (3-(N,N-dimethylamino) propyl-N-ethylcarbodiimide) at a flow rate of 10 μL/min. The antibody at a concentration of 30 μg/mL in 10 mM sodium acetate, pH 5.0, was immobilized at a density of 2000 RU on flow cell 2; flow cell 1 was left blank to serve as a reference surface. All the surfaces were blocked with a 3 min injection at 10 μL/min of 1 M ethanolamine, pH 8.0. GST tagged CK2α at a concentration of 50 μg/mL in 10 mM HEPES, 150 mM NaCl, 3 mM EDTA, 0.005% (v/v) polysorbate 20, pH = 7.4 was captured at a density of 900 RU on flow cell 2. In order to collect kinetic binding data, analytes in 10 mM HEPES, 150 mM NaCl, 3 mM EDTA, 0.005% (v/v) polysorbate 20, 1% DMSO (v/v), 100 μM ATP, pH = 7.4 were injected over the two flow cells at concentrations of 50, 25, 12.5, 6.25, 3.12, 1.56 μM at a flow rate of 25 μL/min and at a temperature of 25 °C. The complex was allowed to associate and dissociate for 120 s. Duplicate injections (in random order) of each sample and a buffer blank were flowed over the two surfaces. Affinities were obtained after treatments (DMSO calibration, blank and references subtractions) using the software Scrubber 2.0c.

### NMR binding experiments

Standard 1D, STD, and WaterLOGSY NMR spectra were acquired at 20 °C with a Bruker 600 MHz NMR spectrometer, equipped with a Z-gradient cryoprobe. WaterLOGSY mixing time was 1.5 s. Both STD and WaterLOGSY spectra were recorded for each sample. WaterLOGSY spectra in the absence of protein receptor were recorded with 300 µM compound to assess the compound solubility. All the NMR experiments were performed at 293 K with excitation sculpting to suppress peaks from water. 1D and STD experiments were performed using identical experimental conditions (spin lock, interscan delays), and parameters for the STD experiments (saturation frequency and saturation time) were identical for all samples. Selective saturation of the protein NMR spectrum was achieved with the offset 2800 Hz upfield from the carrier frequency, and non-saturation control was performed at 15000 Hz downfield. NMR tubes contained 2 μM CK2 with 300 μM of **4**.

### Proximity ligation assay

*In situ* PLAs were performed using a Duolink kit (Olink Bioscience, Uppsala, Sweden) according to the manufacturer’s instructions with few modifications. MCF10A cells were fixed in 4% paraformaldehyde for 10 min. The cells were then permeabilized with 0.1% Triton in Tris-buffered saline (TBS; 50 mM Tris, pH 7.6, 150 mM NaCl) and incubated with 100 mM glycine in phosphate-buffered saline (137 mM NaCl, 2.7 mM KCl, 10 mM Na_2_ HPO_4_, and 1.8 mM KH_2_ PO_4_, pH 7.4) for 20 min. Permeabilized cells were incubated overnight at 4 °C with primary antibodies diluted as follows: mouse CK2α 1:250 and rabbit CK2β 1:50. Cells were washed three times in TBS with 0.05% Tween-20 for 5 min each with gentle agitation. Secondary antibodies conjugated with oligonucleotides, PLA probe anti-mouse MINUS and PLA probe anti-rabbit PLUS, were added to the cells and incubated for 90 min at 37 °C in a humidity chamber. Finally, after ligation and amplification steps, cells were counterstained with the DNA-binding dye Hoechst and Phaloïdine-488 for actin staining (Molecular Probes, Thermo Fisher Scientific, Courtaboeuf, France). Images were observed using a Zeiss Apotome microscope and analyzed using a Zen Pro imaging software (Zeiss, Oberkochen, Germany). Quantification was performed using the BlobFinder software (V3.2, Swedish University of Agricultural Sciences, Uppsala University)^67^. Negative controls were one primary antibody with both of the secondary antibodies.

### Immunoblotting

Cells were lysed in RIPA buffer (10 mM Tris-HCl pH 7.4, 150 mM NaCl, 1% Triton X-100, 0.1% SDS, 0.5% DOC and 1 mM EDTA) containing both protease- and phosphatase-inhibitor cocktails (Sigma-Aldrich; P8340, P2850, P5726). Cell homogenates were quantified using BCA protein Assay kit (Thermo Scientific). SDS-PAGE was performed using pre-cast 4–12% gradient gel (Bio-Rad) and submitted to electrophoresis in NuPAGE buffer (150 V for 75 min). Separated proteins at 20 μg/lane were transferred to PVDF membranes (100 V for 60 min). Blotted membranes were blocked during 1 h at room temperature with saturation buffer (1% BSA in Tris Buffer Saline 10 mM, Tween 0.1% (TBST)), and then incubated with primary antibody diluted in saturation buffer, for 2 h or overnight. After 3 washes with TBST, secondary antibodies were added for 1 h followed by 3 more washes with TBST. Luminata Forte Western HRP substrate (Millipore) was added and membranes were read with Fusion Fx7 (PerkinElmer). GAPDH or HSP90 was used as loading control. Images were analyzed and band intensities were quantified using ImageJ software. Primary antibodies were GAPDH antibody from Ambion, PARP and HSP90 antibodies from Cell Signaling, survivin antibody from Novus biologicals, p21 antibody from Santa Cruz Biotechnologies, p21-(phospho-Thr145) antibody from Abcam. Secondary antibodies were peroxidase-conjugated affinity pure Goat anti-rabbit IgG (#111035003) and peroxidase-conjugated affinity pure Goat anti-mouse IgG (#115035003) from Jackson Immuno Research.

### Live cell tracking

Cells grown on 96-well flat-bottomed plates (Corning Falcon) were tracked using an Essen IncuCyte Zoom live-cell microscopy instrument, an automated live cell imager with high-throughput capabilities and built-in data analysis. Experiments were conducted at 37 °C and 5% CO_2_. The software incorporated into the IncuCyte Zoom was used to analyze the images.

#### Cell proliferation

MCF10A and MDA-MB231 cells were seeded into 96-well plates at a density (1 × 10^4^ and 2 × 10^4^ cells/well, respectively) and allowed to attach overnight. Bright field images were captured every 3 h for the duration of the experiment. The detection software was calibrated specifically for MCF10A and MDA-MB231 to ensure accurate distinction of cells from empty space. Cell proliferation data were obtained by the cell confluence increment in each of the treatments and expressed as percentage relative to that of control cells.

#### Cell migration

Cells seeded at equal density were allowed to grow to confluence overnight. The Essen Bioscience WoundMaker^TM^, an accessory for the IncuCyte Zoom®, was utilized to create Scratch-wounds of a standardized width (~600 μm). Cells were treated with the indicated inhibitors and imaged every 15 min for 44 h and the percentage of wound confluence was analyzed.

#### Cell death

Cells plated at equal density (2 × 10^4^ cells/well) were treated with the indicated inhibitors in cell culture medium containing 0.5 μg/ml Propidium iodide (Sigma-Aldrich). Images of PI-stained red fluorescent cells were captured every 3 h for the duration of the experiment. For apoptosis detection, 50 μL/mL fluorochrome-conjugated Annexin V (Molecular Probes) was added to the medium together with Propidium iodide and apoptotic cell numbers were calculated.

### Crystallography

CK2α was expressed and purified for crystallization as previously published^37^. CK2α_KA at 5 mg/mL in 20 mM Tris, pH 8.0, 350 mM NaCl, 1 mM DTT, and 25 mM ATP was crystallized with 112.5 mM MES pH 6.5, 35% glycerol ethoxylate and 180 mM ammonium acetate in a 1:1 ratio with a total volume of 2 μL by the hanging drop vapour-diffusion method. The compounds were soaked at at 10 mM into these crystals for 15–20 h in 107 mM MES pH 6.5, 35% glycerol ethoxylate and 1.04 M ammonium acetate after which the crystals were cryo-cooled in liquid nitrogen for data collection. The crystals were cryo-cooled in liquid nitrogen in the same solution for data collection.

X-ray diffraction data was collected at the Diamond synchrotron radiation source, then processed using the pipedream package by Global Phasing Ltd; structures were solved by using programs from the CCP4 package. Models were iteratively refined and rebuilt by using AutoBuster and Coot programs. Ligand coordinates and restraints were generated from their SMILES strings using the Grade software package. All coordinates have been deposited to Protein Data Bank and accession numbers, data collection, refinement statistics, crystallisation and soaking conditions are shown in Table S1.

## Supplementary information

Supplementary Information

## Data Availability

Custom scripts for interface fumigation were written in the ICM scripting language and are available from the authors upon request.
