# Supplementary material for: Discovery of holoenzyme-disrupting chemicals as substrate-selective CK2 inhibitors
Source: Sci Rep. 2019 Nov 4;9:15893. doi: 10.1038/s41598-019-52141-5 (PMC6828666; doi:10.1038/s41598-019-52141-5)
Supplement: Supplementary file 1 — Supplementary Information [file 41598_2019_52141_MOESM1_ESM.docx]

*Supporting Information*

**Discovery of holoenzyme-disrupting chemicals as substrate-selective CK2 inhibitors**

Irina Kufareva^1*^, Benoit Bestgen^2,3,4,5*^, Paul Brear^6^, Renaud Prudent^4,7^, Béatrice Laudet^4^, Virginie Moucadel^4,8^, Mohamed Ettaoussi^2^, Celine F. Sautel^4,9^, Isabelle Krimm^10^, Matthias Engel^3^, Odile Filhol^4^, Marc Le Borgne^2^, Thierry Lomberget^2^, Claude Cochet^4#^, Ruben Abagyan^1#^

**Table of contents**

**1. Supplementary Figures and Table**

**Fig. S1.** CK2 inhibition by compound **1** is not mediated by aggregation and is reversible.

**Fig. S2.** K_i_ and K_i_’ value determination from enzymatic kinetic characterization of CK2 inhibition by compound **1**.

**Fig. S3.** Compound **1** inhibits CK2β-dependent phosphorylation of Olig2 transcription factor and CK2β-dependent autophosphorylation.

**Fig. S4.** Kinase selectivity profile of compound **1** on a panel of 45 different protein kinases.

**Fig. S5.** Binding mode of compound 1 predicted by ICM virtual docking.

**Fig. S6.** Compounds **1**, **4** and **6** selectively inhibit the phosphorylation of CK2β-dependent peptide substrate.

**Fig. S7.** Binding kinetics of compounds **2-6** by surface plasmon resonance.

**Fig. S8.** NMR-based investigation of the binding of compound **4** to CK2α.

**Fig. S9.** Compound **6** induces cell growth inhibition.

**Fig. S10.** Effect of compound **6** on cell migration.

**Fig. S11.** Effects of compound **4** on cell death and proliferation of MDA-MB231 cells.

**Fig. S12.** Compound **6** induces rapid apoptosis in MDA-MB231 cells.

**Fig. S13.** αD loop transition upon compound **6** binding.

**Fig. S14.** Interface fumigation.

**Fig. S15.** Synthesis of compounds **2-6**.

**Fig. S16.** Uncropped versions of blots shown in main text figures 4a, 5a-d, and supplementary figures S3a, c, d and S9b.

**Table S1.** Protein Data Bank accession numbers, data collection, refinement statistics, crystallisation and soaking conditions for co-crystal structures of CK2α with **4** (CCH507) and **6** (CCH503).

**2. Supplementary Material and Methods**

Synthetic route and chemistry procedures to synthesize compounds **2-6**.

Supplementary Figures and Table

**
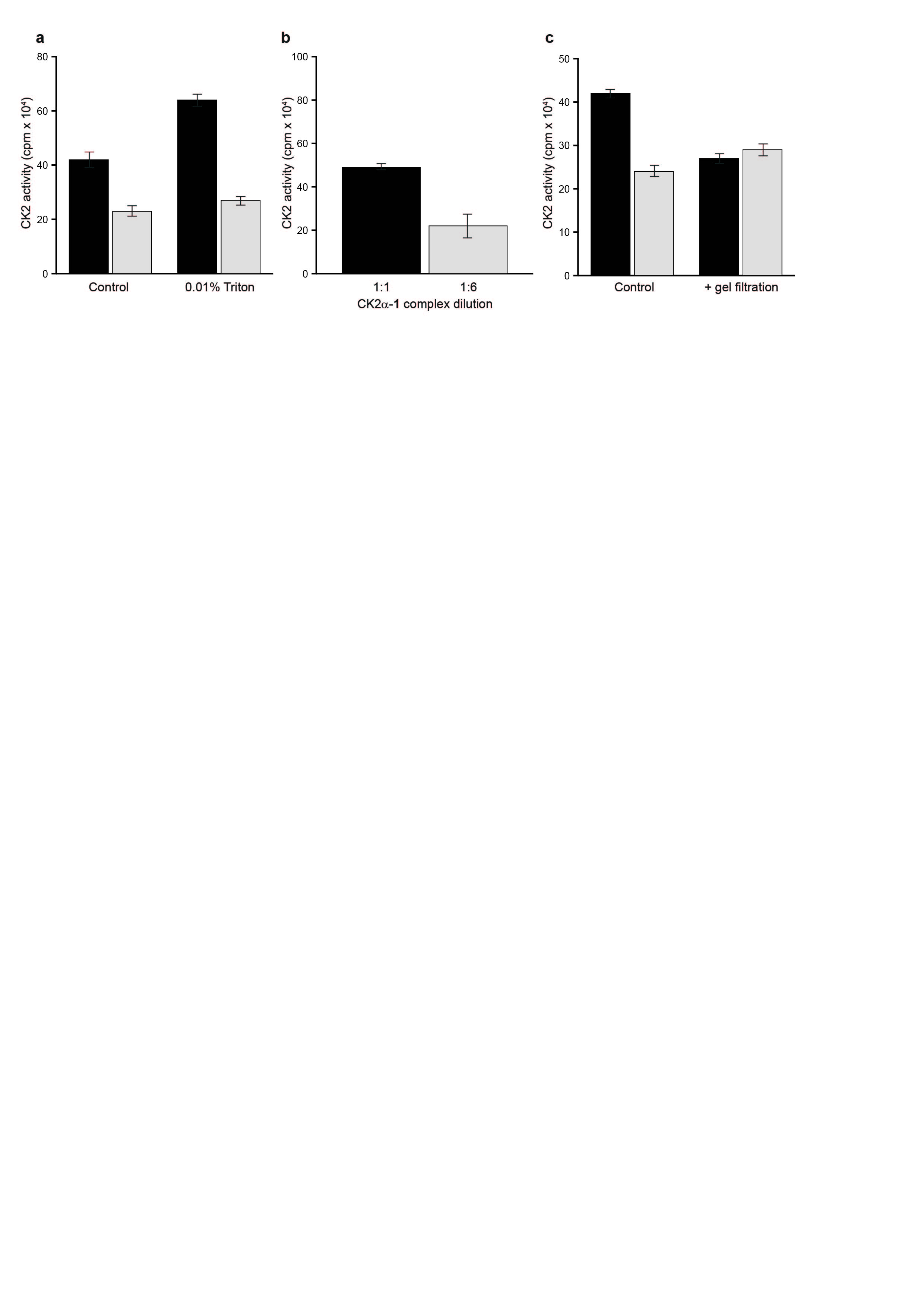
**

**Fig. S1. CK2 inhibition by compound 1 is not mediated by aggregation and is reversible. (a)** CK2 inhibition by compound **1** is insensitive to Triton X-100. CK2α (200 nM) was incubated with 200 nM CK2β in the absence (black bar) or presence (grey bar) of 50 μM of compound **1**. Incubations were performed in the absence or presence of 0.01% Triton X-100. CK2 activity was determined using saturating concentrations of the CK2β-dependent peptide substrate (600 μM) and ATP (100 μM); **(b)** CK2 inhibition by compound **1** is reversible upon dilution. CK2α (80 nM) was incubated with 280 nM CK2β in the absence or presence (grey bar) of 50 μM of compound **1**. CK2 activity was determined in the mixtures either undiluted or diluted 1/6 in kinase buffer using saturating concentrations of CK2β-dependent peptide substrate (600 μM) and ATP (100 μM). The results are expressed as % of inhibition of CK2 activity in the absence or presence of compound **1**; **(c)** CK2 inhibition by compound **1** is reversible upon gel filtration. CK2α (200 nM) was incubated in the absence (black bar) or presence (grey bar) of 100 μM of compound **1**. Aliquots of the mixtures were saved (Control); the remaining mixture was subjected to a rapid filtration (GF) on Bio-Spin 6 columns. CK2 activity in the control and GF samples was determined in the presence of CK2β using saturating concentrations of CK2β-dependent peptide substrate (600 μM) and ATP (100 μM). Error bars represent the SEM of two biological replicates derived from technical triplicates.

**
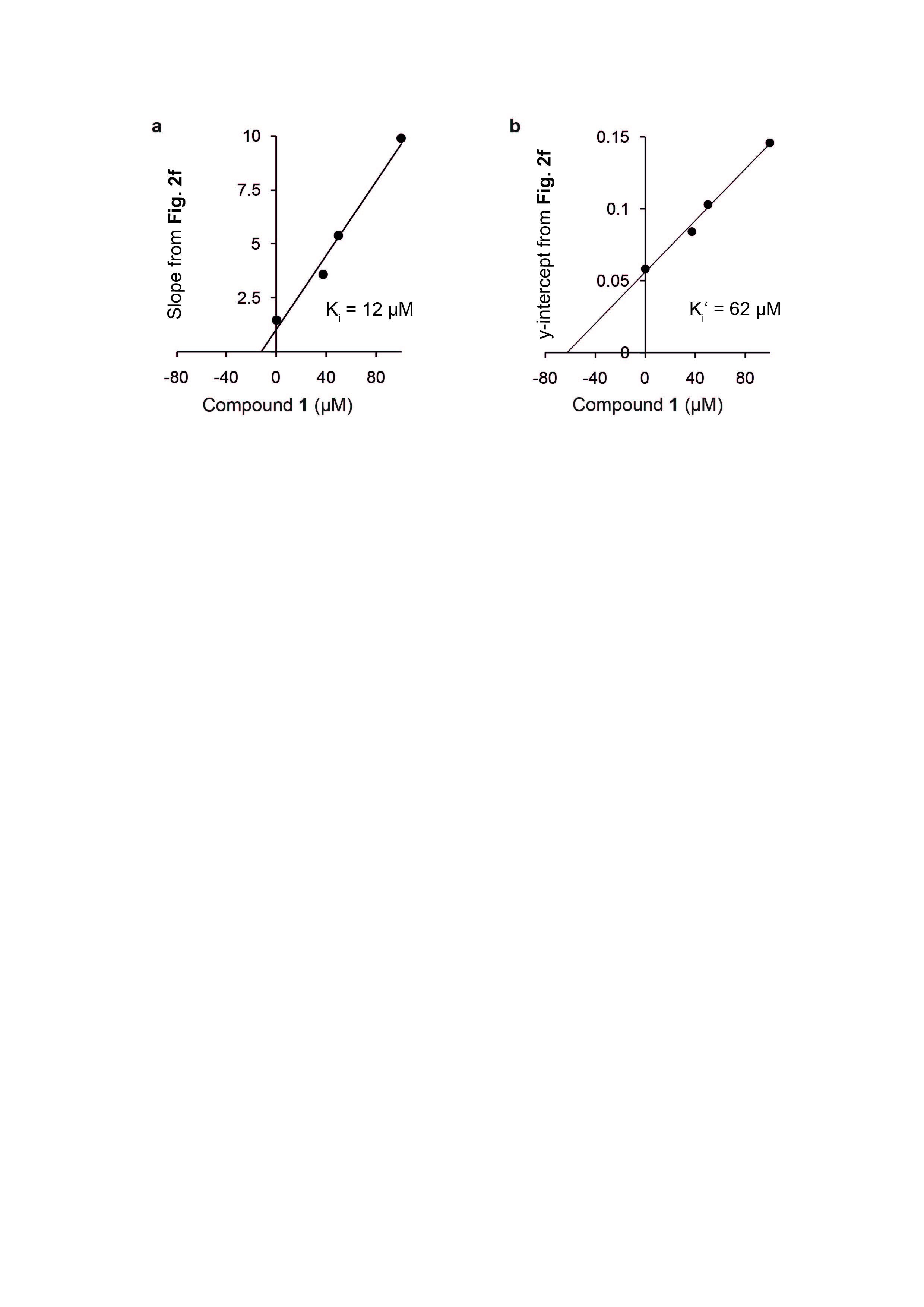
**

**Fig. S2. K_i_ and K_i_’ value determination from enzymatic kinetic characterization of CK2 inhibition by compound 1.** **(a)** K_i_ value has been calculated from linear regression analysis of the slopes (α/V_max_) of the Lineweaver-Burk double reciprocal plots of Fig 2f. **(b)** K_i_’ value has been calculated from linear regression analysis of the y-intercept (α’/V_max_) of Lineweaver-Burk double reciprocal plots of Fig 2f.

**
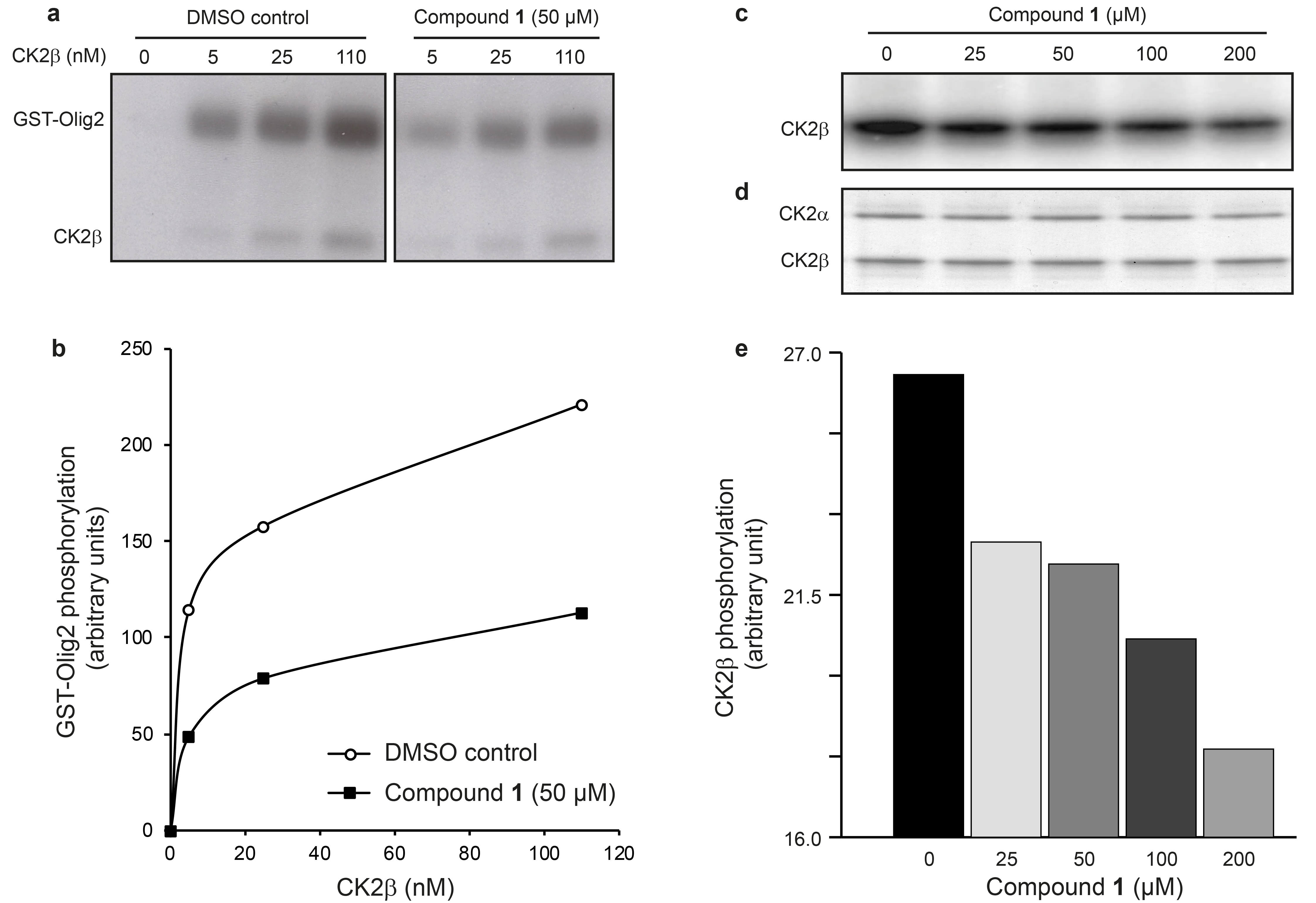
**

**Fig. S3. Compound 1 inhibits CK2β-dependent phosphorylation of Olig2 transcription factor and CK2β-dependent autophosphorylation. (a)** CK2α (200 nM) was incubated with increasing concentrations of CK2β in the absence or presence of compound **1** (50 μM) followed by the addition of GST-Olig2 (3.7 μg), [γ^32^P]-ATP (100 μM, 1 μCi) and MgCl_2_ (10 mM). Samples were analyzed by SDS PAGE and subjected to autoradiography. Left and right parts of the image are from the same gel (see Supplementary Fig. S16). **(b)** ^32^P incorporated into GST-Olig2 was determined by densitometry scanning and is expressed as arbitrary units; **(c)** CK2β (300 nM) was incubated with increasing concentrations of compound **1**, 10 μM [γ^32^P] ATP and 10 mM MgCl_2_. Autophosphorylation was initiated by the addition of 300 nM CK2α. Phosphorylated proteins were separated by SDS/PAGE and analyzed by autoradiography **(c)** or Coomassie Blue staining **(d)**; ^32^P incorporation into CK2β was quantified and expressed as arbitrary units **(e)**. Uncropped blots are shown in Supplementary Fig. S16.

**
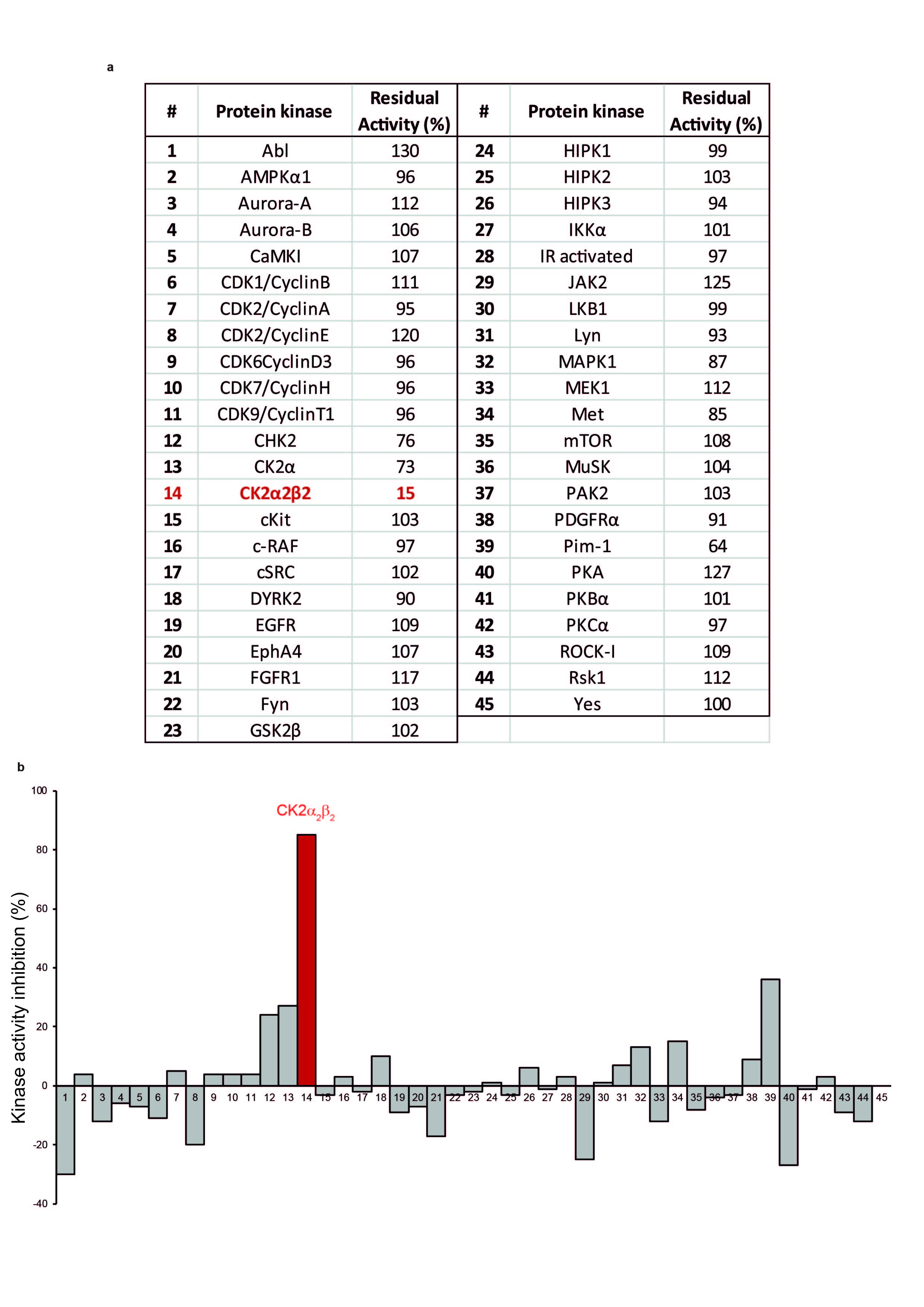
**

**Fig. S4. Kinase selectivity profile of compound 1 on a panel of 45 different protein kinases.** The assays were performed at 10 μM ATP in the presence of 100 μM compound **1** using the Upstate Kinase Profiler Panel Service (Millipore). Residual activity in presence of 100 μM compound **1** are depicted in **(a)** and a graphical representation of the inhibition percentage in the presence of 100 μM compound **1** is shown in **(b)**.

**
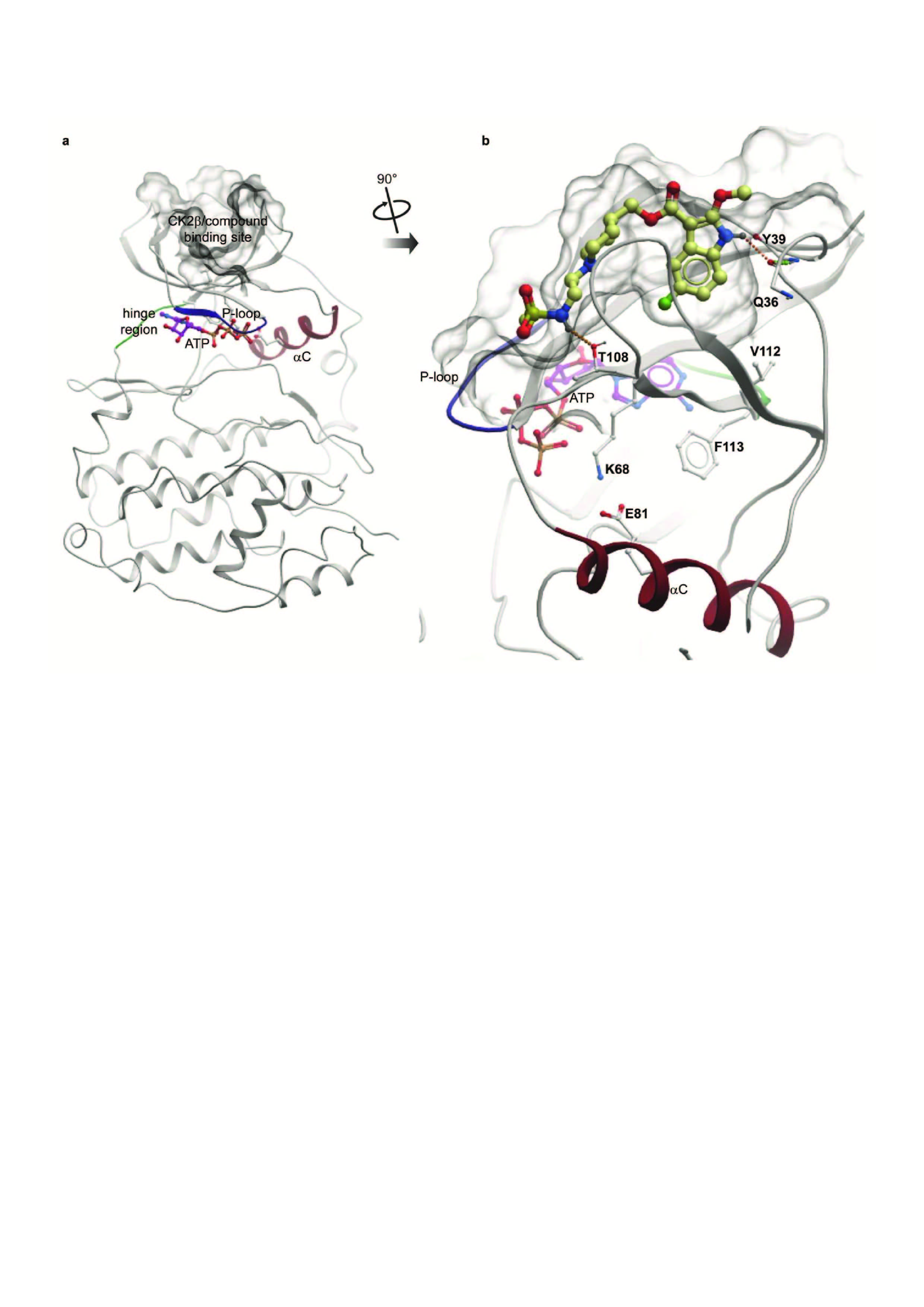
**

**Fig. S5.** **Binding mode of compound 1 predicted by ICM virtual docking.** **(a)** Overall architecture and compound/CK2β binding site; **(b)** Predicted pose of compound **1**.

**
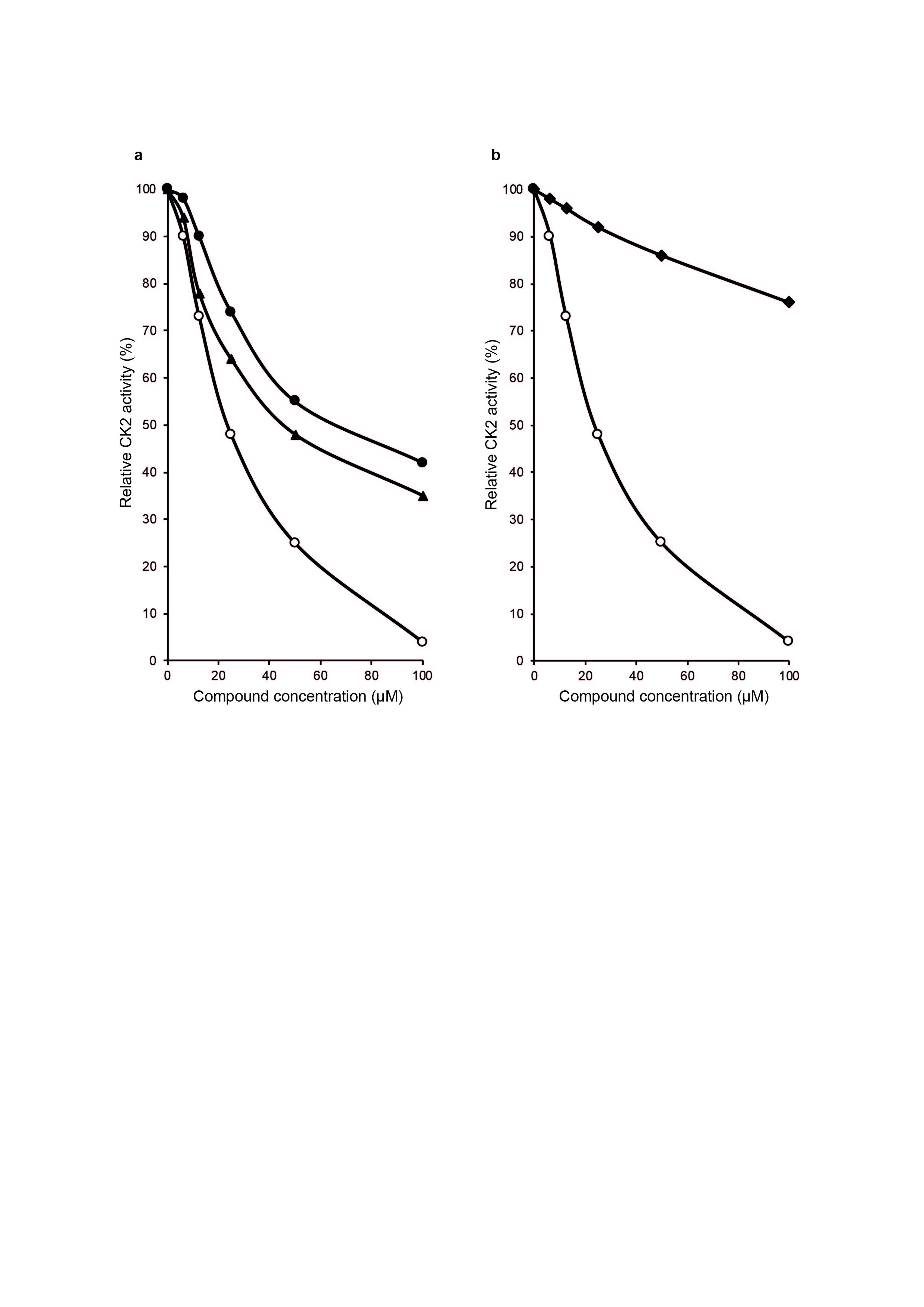
**

**Fig. S6. Compounds 1, 4 and 6 selectively inhibit the phosphorylation of CK2β-dependent peptide substrate.** **(a)** Dose-dependent inhibition of CK2. CK2α (40 nM) was incubated with increasing concentrations of compound **1** (●), **4** (▲) or **6** (○) in the presence of 20 nM CK2β. CK2 activity was determined with a saturating concentration of CK2β-dependent peptide substrate. **(b)** Differential effects of **6** on the phosphorylation of CK2 peptide substrates. CK2α (40 nM) was incubated with increasing concentrations of **6** in the presence of 20 nM CK2β. CK2 activity was determined with a saturating concentration of CK2β-independent peptide substrate (⯁) or CK2β-dependent peptide substrate (○). Data are representative curves of two biological replicates, derived from technical duplicates.

**
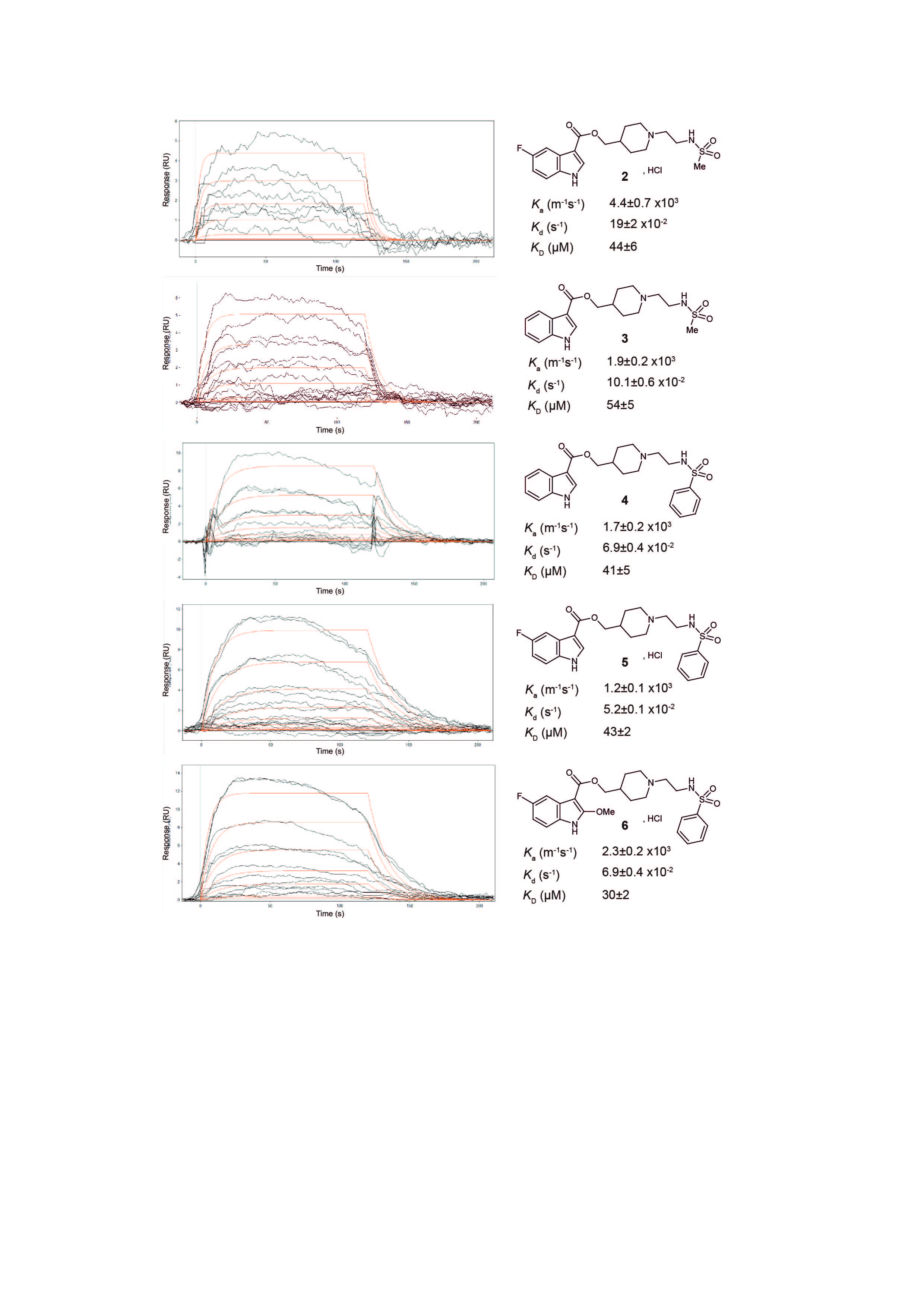
**

**Fig. S7. Binding kinetics of compounds 2-6 by surface plasmon resonance.** For each compound, sensorgrams (black line) and global fits (orange line) are depicted with the corresponding chemical structure and the fitted kinetics parameters. Experiments show representative data of two identical repeats.


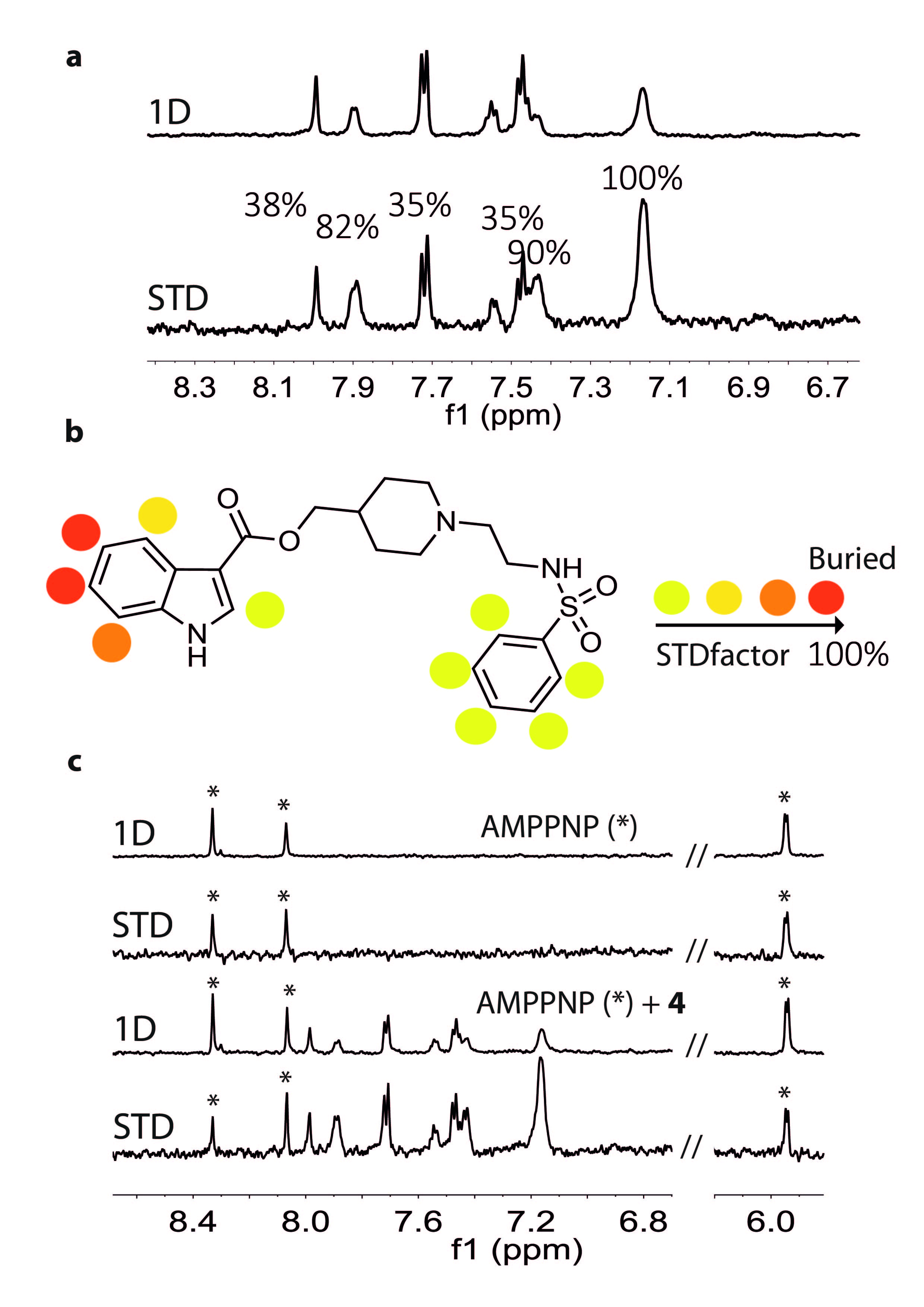


**Fig. S8.** **NMR-based investigation of the binding of compound 4 to CK2α. (a)** NMR-STD epitope mapping observed for compound **4**. The STD factors (expressed as percentage in **(a)** discriminate the solvent-exposed protons of compound **4** (weak STD factor values) from the buried protons of compound **4** when bound to CK2α (STD factor values 100%), suggesting a particular orientation for the indole moiety. The latter is observed as the most buried part of the inhibitor. **(b)** Schematic representation of the STD factor values on the chemical structure of compound **4**. **(c)** NMR-STD competition experiment between AMPPNP, an ATP analogue, and compound **4**, for CK2α binding. The STD signals of AMPPNP, observed upon CK2α binding (upper panel), are not modified in the presence of compound **4** (lower panel). Both compounds bind simultaneously to CK2α.


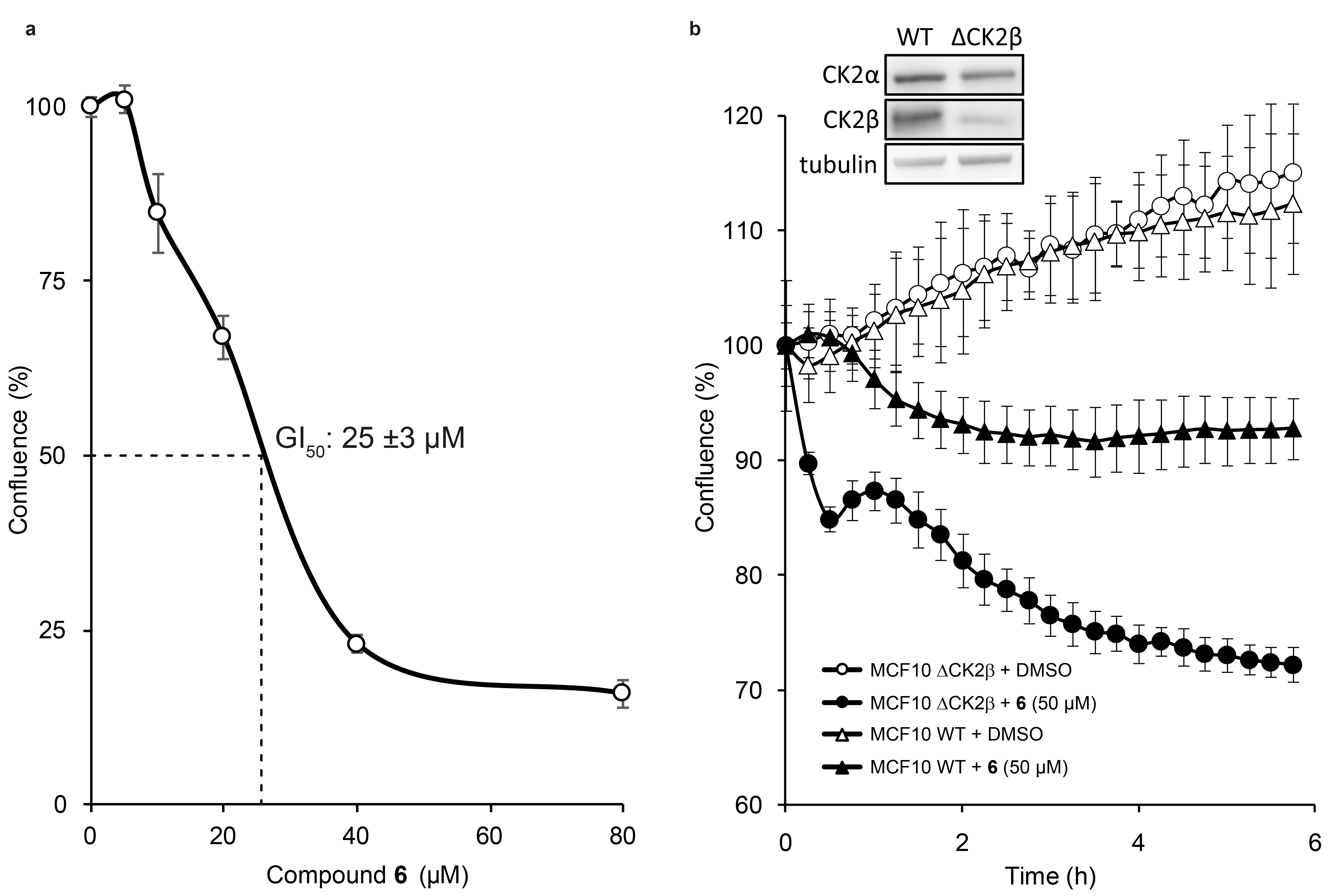


**Fig. S9. Compound 6 induces cell growth inhibition. (a)** MCF10A cells were incubated with increasing concentrations of **6** for 24 h. **(b)** Inset: western blot analysis of CK2 subunits in parental MCF10A cells (WT) or CK2β knockdown MCF10A cells (∆CK2β). Uncropped blots are shown in Supplementary Fig. S16. MCF10A WT (**△**,▲) or MCF10A ∆CK2β (**○**,**●**) were incubated with DMSO (**△**,**○**) or 50 μM **6** (▲,●) for the indicated time. Cell growth was monitored using an Essen IncuCyte Zoom live-cell microscopy incubator. Images were captured every 15 min for the duration of the experiment. Error bars represent the SEM of two biological replicates derived from technical triplicates.

**
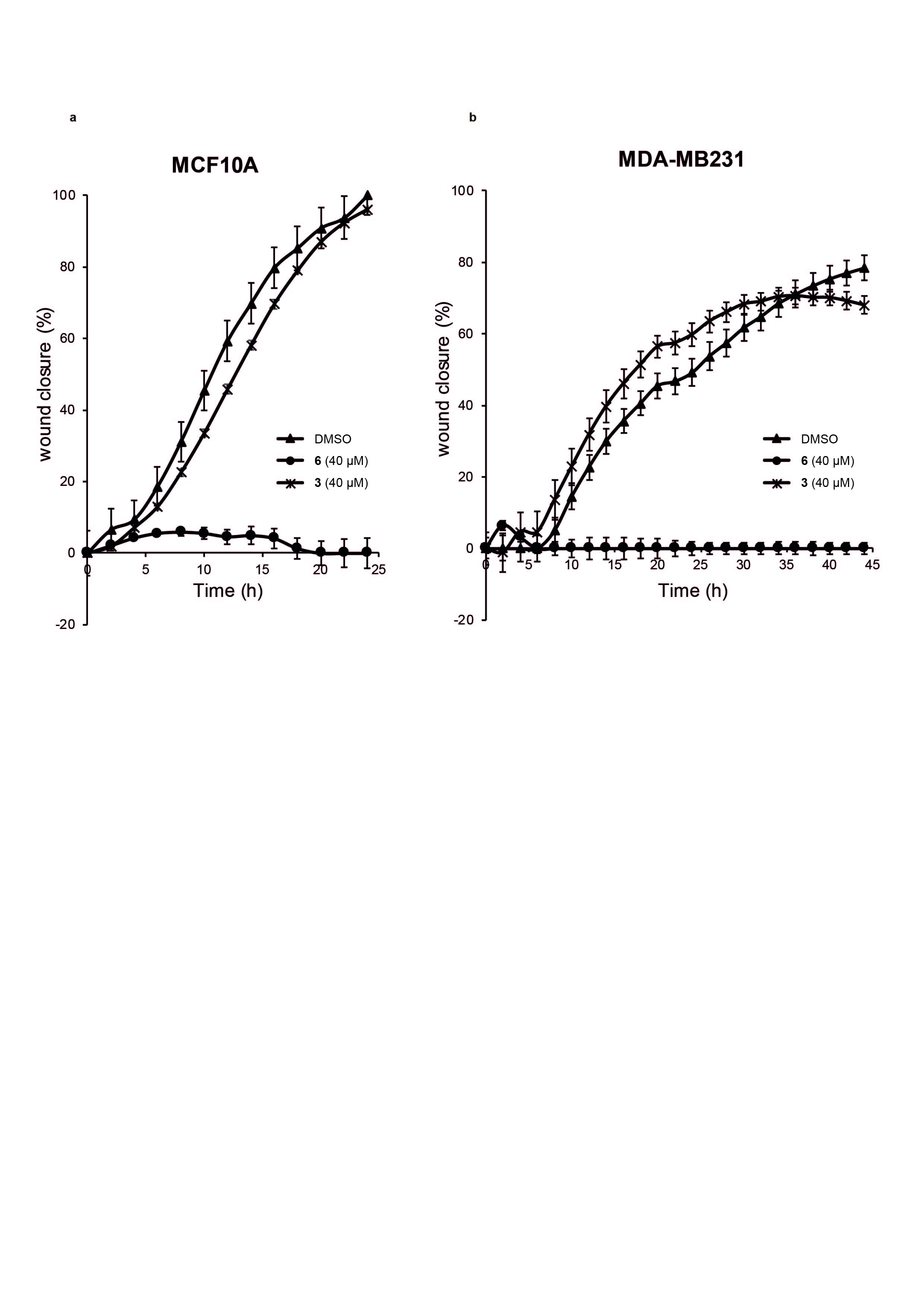
**

**Fig. S10. Effect of compound 6 on cell migration.** MCF10A cells **(a)** or MDA-MB231 cells **(b)** were allowed to grow to confluence overnight. Wounds of a standardized width were created using the Essen Bioscience WoundMakerTM. Cells were then incubated with DMSO (▲) or 40 μM compound **6** (●) or the inactive analog **3** (**x**) for the indicated time. Wound closure was monitored using the Essen IncuCyte Zoom live-cell microscopy incubator. Images were captured every 2 h for the duration of the experiment. Error bars represent the SEM of two biological replicates derived from technical triplicates.

**
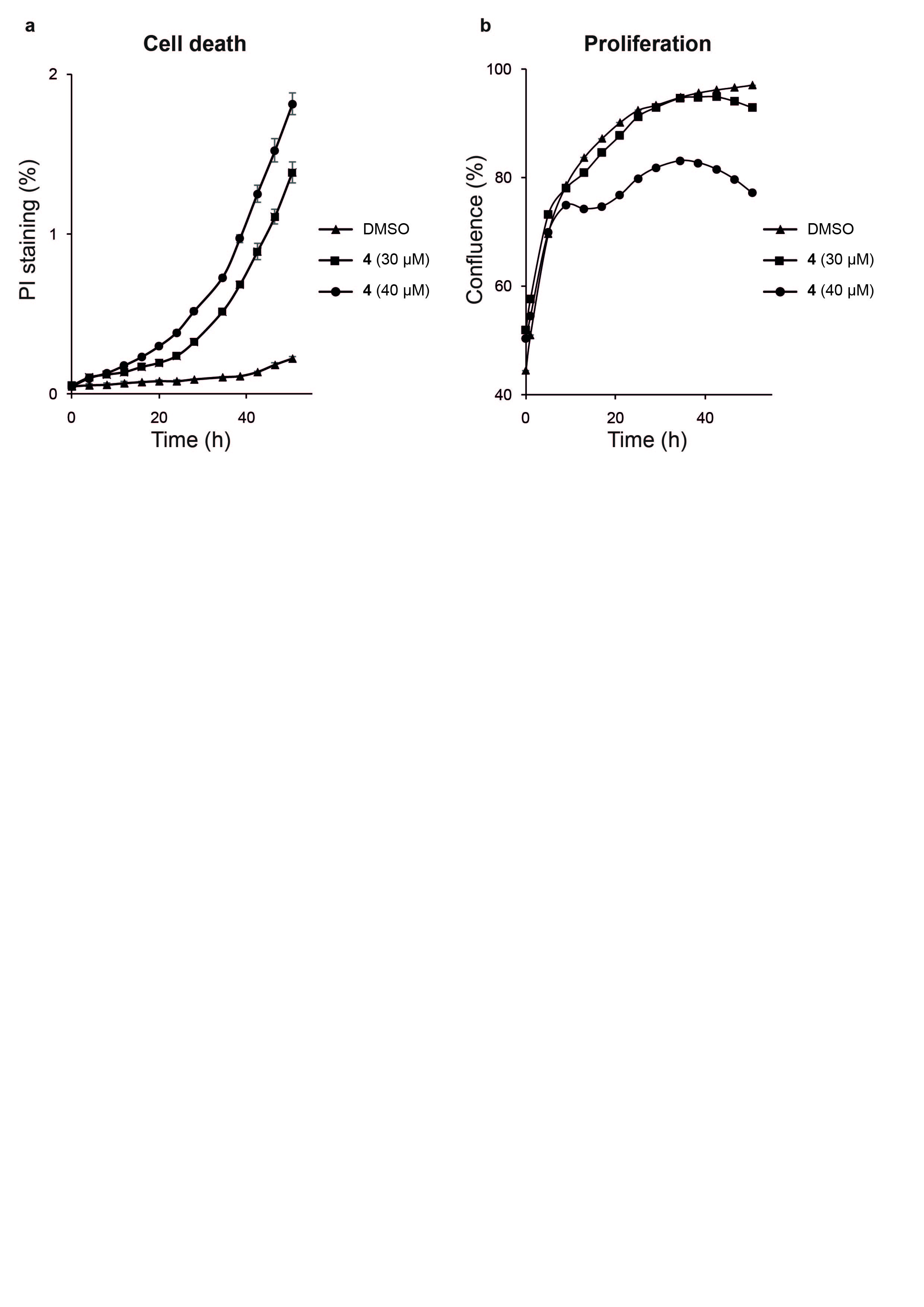
**

**Fig. S11. Effects of compound 4 on cell death (a) and proliferation (b) of MDA-MB231 cells.** Cells were treated with DMSO (▲), or 30 μM (■) or 40 μM (●) of **4**. Cells were tracked using an Essen IncuCyte Zoom live-cell microscopy incubator and images were captured every 3 h for the duration of the experiments. Data are representative of two biological replicates, derived from technical triplicates.

**
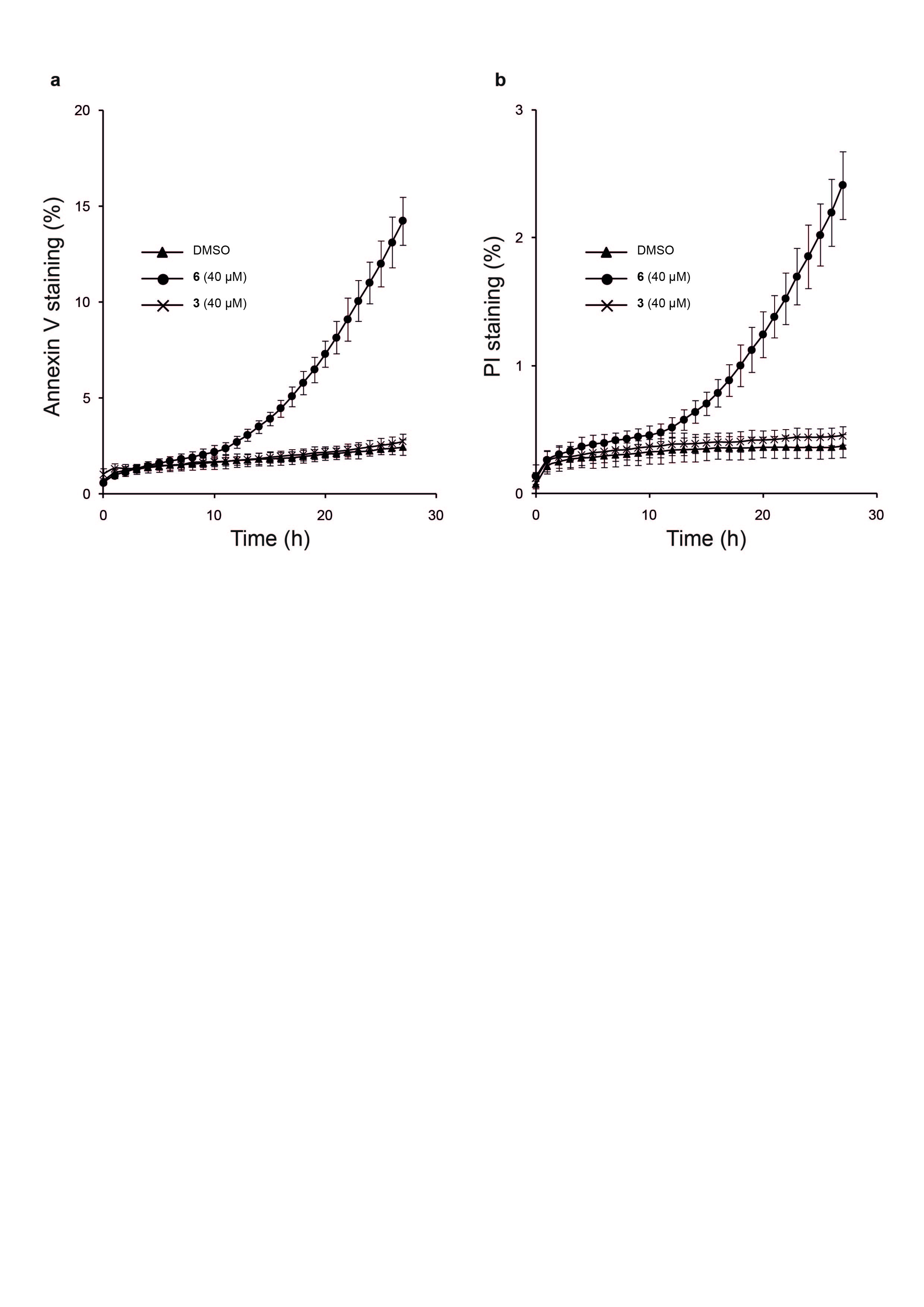
**

**Fig. S12. Compound 6 induces rapid apoptosis in MDA-MB231 cells.** Time course analysis of MDA-MB231 cells reveals that **6** induces cell apoptosis in a time-dependent manner. Cells were treated with DMSO (▲), or 40 μM **6** (●) or 40 μM of the inactive analog **3** (**X**). Cell viability was assessed by Annexin V staining **(a)** or PI staining **(b)** using the Essen IncuCyte Zoom live-cell microscopy incubator. Images were captured every hour for the duration of the experiment. Error bars represent the SEM of two biological replicates derived from technical triplicates.

**
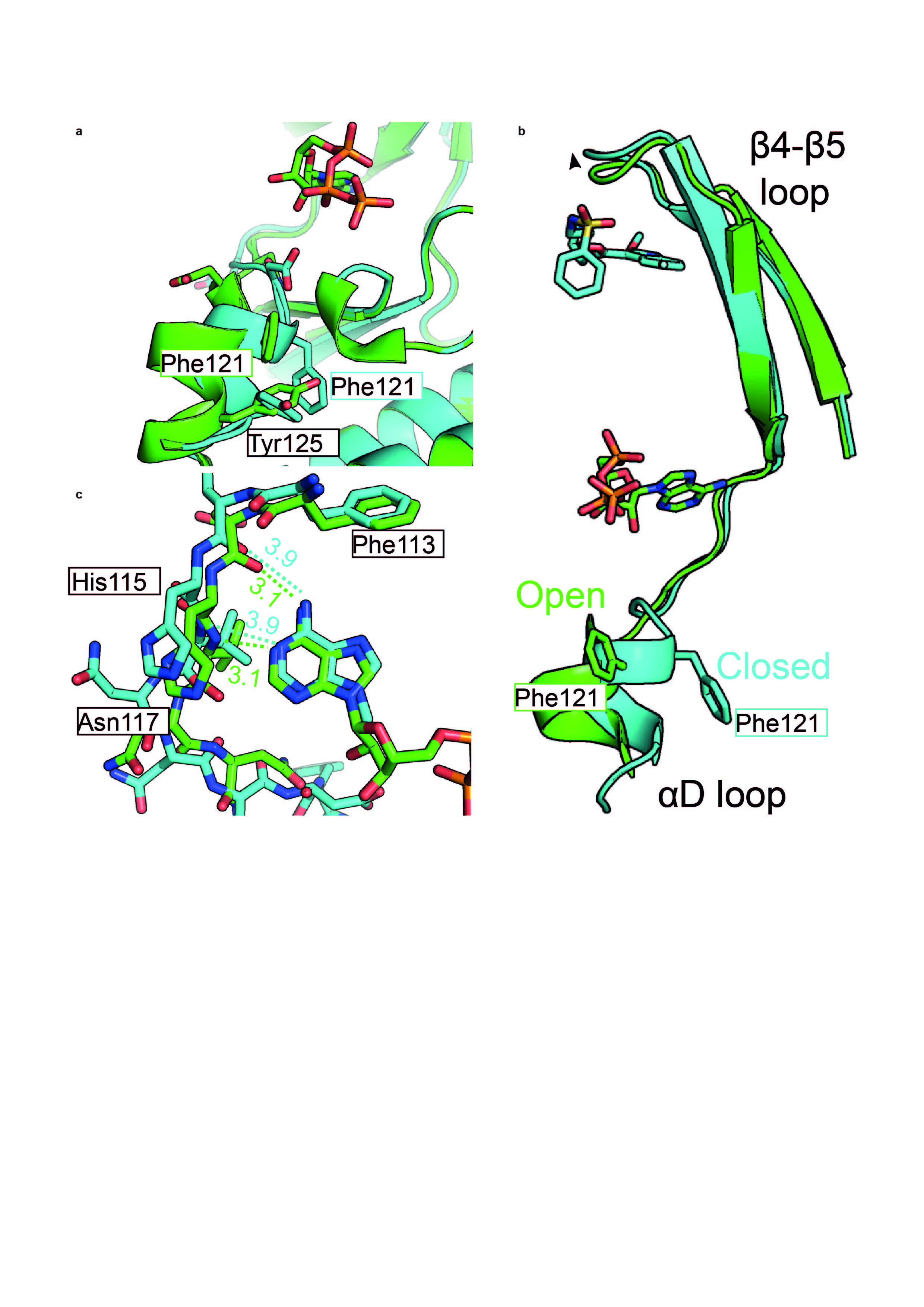
**

**Fig. S13. αD loop transition upon compound 6 binding. (a)** The movement of the αD loop upon the binding of **6** to the interface site. The apo structure with the αD loop in the closed position in shown green (pdb:5CU6). The structure with **6** bounds where the αD loop in the closed position is shown in blue (pdb:6FVG). **(b)** Highlighting just the link between the β4-β5 loop and the αD loop. The linked movement of both regions upon the binding of **6** can be clearly seen. The apo structure is shown in green and the structure with **6** bound is shown in blue. **(c)** The movement of the hinge region upon the binding of **6**. The structure with **6** bound is shown in green and the apo structure with ATP bound is shown in green. All distances are shown in Angstroms.

**
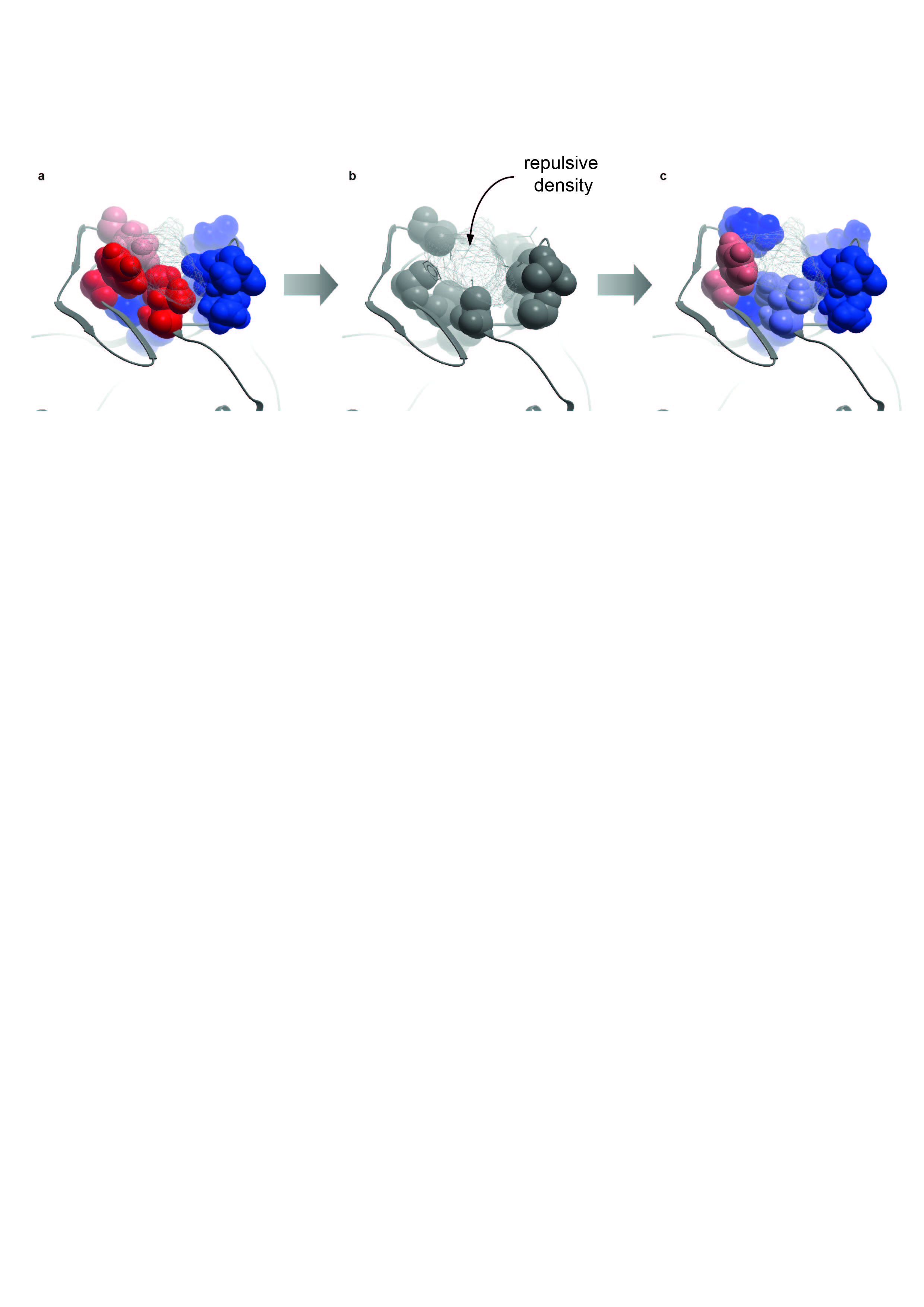
**

**Fig. S14. Interface fumigation. (a)** the original X-ray structure; **(b)** the result of simultaneous conversion of all interface residues to Ala: the “largest accessible pocket” density is generated; **(c)** a “druggable” interface conformation obtained by Monte Carlo simulation in the presence of the density. Coloring of the residues indicates the degree of their intrusion into the density (blue – low, red – high).

**Fig. S15. Synthesis of compounds 2-6.** Reagents and conditions: (a) For **8a** (R=Me): *N*-(2-chloroethyl)methanesulfonamide 30 °C, 48 h, 30%. For **8b**, (R=Ph): *N*-(2-chloroethyl)benzenesulfonamide, *i*-Pr_2_EtN, acetonitrile, 85 °C, 3h, 76%. b) 1) Trifluoroacetic anhydride, DMF, 0 °C then 30 °C, 0.5-3 h. 2) Solid filtration then 20% aqueous NaOH, 50 °C, 18 h. 75% for **10a** (R^5^=H); 81% for **10b** (R^5^=F). c) 1) Indole **10**, SOCl_2_, ε DMF or not, CH_2_Cl_2_, 35 °C, 24-48 h. 2) Solvent evaporation then CH_2_Cl_2_, alcohol **8**, 35 °C, 24 h, 30% for **2**, 50% for **3**, 76% for **4**, 63% for **5**. d) 1) *N*-chlorosuccinimide, CHCl_3_, reflux 4 h, solvent evaporation. 2) MeOH, 65 °C, 12 h, 56%.

**
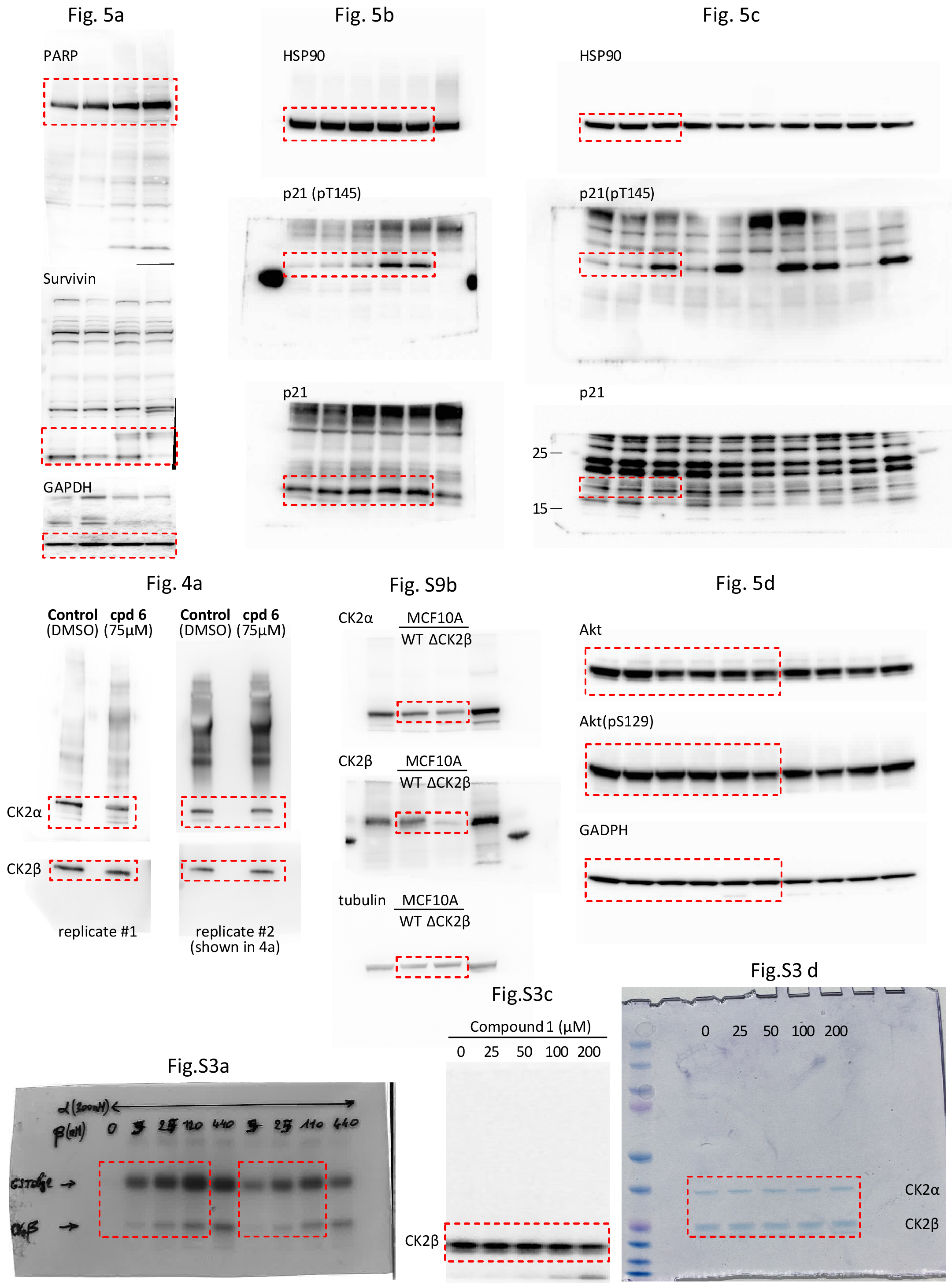
**

**Fig. S16.** Uncropped versions of blots shown in main text figures 4a, 5a-d, and supplementary figures S3a, c, d and S9b.

**Table S1. Protein Data Bank accession numbers, data collection, refinement statistics, crystallisation and soaking conditions for co-crystal structures of CK2α with 4 (CCH507) and 6 (CCH503).**

| **Structure** | **CCH503 CK2a 007** | **CCH507 CK2a 002** |
| --- | --- | --- |
| PDB code | 6FVF | 6FVG |
| Ligand | CCH503 | CCH507 |
| Protein | CK2α_KA | CK2α_KA |
|  |  |  |
| Data Collection: |  |  |
| Beamline | Diamond Light source IO2 | Diamond Light source IO4-1 |
| Wavelength | 0.9795 | 0.9282 |
| Resolution range | 58.53 - 1.47 (1.510 - 1.470) | 56.10 - 1.60 (1.640 - 1.600) |
| Space group | P 1 21 1 | P 1 21 1 |
| Cell (a b c) | 58.99 45.25 62.75 | 60.18 45.93 63.71 |
| Cell (alpha beta gamma) | 90.00 111.15 90.00 | 90.00 111.22 90.00 |
| Total reflections | 169330 (12293) | 140961 (10561) |
| Unique reflections | 52646 (3864) | 43028 (3180) |
| Multiplicity | 3.2 (3.2) | 3.3 (3.3) |
| Completeness (%) | 99.8 (99.6) | 99.9 (99.8) |
| Mean I/sigma(I) | 9.0 (1.1) | 9.2 (1.3) |
| R-merge | 0.055 (1.04) | 0.049 (0.73) |
| R-pim | 0.037 (0.68) | 0.031 (0.47) |
| CC-half | 0.994 (0.61) | 0.997 (0.74) |
|  |  |  |
| Refinement: |  |  |
| R-factor / Rfree | 0.200/0.222 | 0.206/0.229 |
| Number of total atoms | 3023 | 3061 |
| atoms for ligands | 45 | 62 |
| atoms for waters | 163 | 191 |
| Number of polymer residues | 327 | 327 |
| Average/Wilson B-factor | 34.1/21.0 | 36.5/25.2 |
| B-factor for ligands | 54.8 | 70.6 |
| B-factor for solvent | 37.5 | 38.4 |
| RMS(bonds) | 0.01 | 0.01 |
| RMS(bond angles) | 0.99 | 0.92 |
| RMS(dihedral angles) | 3.18 | 2.7 |
| Crystallisation conditions | 112.5mM Mes pH 6.5, 35% glycerol ethoxylate, 180 mM ammonium acetate | 112.5mM Mes pH 6.5, 35% glycerol ethoxylate, 180 mM ammonium acetate |

Supplementary Materials and Methods

**Chemistry**

Synthetic route

The synthesis of analogues **2-6** is based on a convergent approach, involving an esterification step between piperidine alcohols **8** and indole 3-carboxylic acids **10**.

Preparation of piperidin-4-yl-methanol **7** was realized by reduction of ethyl isonipecotate using lithium aluminum hydride in tetrahydrofuran, according to a literature reference.^[[1]](#footnote-1)^ The *N*-alkylation of the piperidine heterocycle of **7** was then carried out using two different reagents, depending of the desired sulfonamide function (either methyl or phenyl), leading to building blocks **8a**,**b**.

The preparation of indole 3-carboxylic acids **10** relies on a two steps sequence starting from indoles **9**, combining an acylation reaction with trifluoroacetic anhydride and a basic hydrolysis of the 3-trifluoroacetate intermediate.^[[2]](#footnote-2)^

The esterification step of indole 3-carboxylic acids **10a**,**b** was realized through their conversion in acyl chlorides, followed by *in situ* reaction with the alcohols **8a**,**b**.

Functionalization with a methoxy group at position 2 of the indole core relies on a one-step protocol: *in situ* chlorination of this position, followed by nucleophilic substitution of the halide with methanol, used as the reaction solvent.^[[3]](#footnote-3)^

General chemistry procedures

All commercially available chemicals and solvents were purchased from Sigma-Aldrich, Acros Organics, Fischer scientific and Alfa Aesar. They were used without further purification, unless specified and the reaction solvents are of anhydrous grade (Acros ACROSEAL^TM^). Reactions were carried out in oven-dried glassware, under a positive pressure of argon and monitored by thin layer chromatography, after UV visualizations (at 254 and 366 nm) or chemical staining (ninhydrin or anisaldehyde solutions). Product purification by flash column chromatography was performed using silica gel Merck Kieselgel 60 Å (40–63 µm); solvent composition is indicated under parentheses. Thin Layer Chromatographies (TLC) (thickness: 200 μm) were purchased from Merck.

^1^H NMR spectra were obtained on Bruker ALS300 and DRX300 (at 300 MHz), and DRX400 Bruker (at 400 MHz) spectrometers, using the residual signal of deuterated NMR solvent as internal reference.^[[4]](#footnote-4)^ Chemical shifts are expressed in parts per million (ppm), multiplicity of the signals are indicated by lower-case letters (singlet s, doublet d, triplet t, quadruplet q, multiplet m, broad singlet br s, doublet of triplet dt, triplet of doublet td, triplet of triplet tt), coupling constants are expressed in Hertz (Hz), and deuterated solvents are either dimethylsulfoxide-d_6_ or CDCl_3_. ^13^C NMR spectra were obtained on Bruker DRX300 (at 75 MHz) and DRX400 Bruker (at 100 MHz). Melting points are measured on a Büchi B-540 melting point apparatus and are uncorrected. HPLC/MS analyses were carried out on an Agilent 1290 Infinity system with a binary pump, degasser, autosampler, thermostated column compartment, 1260 Infinity Diode Array Detector and 6120 single quadrupole mass spectrometer equipped with an ElectroSpray Ionization source (low resolution mass spectra were recorded at the positive mode (ESI+)). The entire system was controlled by ChemStation (Agilent Technologies, Rev B.04.02 SP1). The column was an Agilent Poroshell 120 SB-C18, 2.7 µm, 2.1x50 mm. The samples were analyzed in the positive ion mode of the Electron Spray Ionization (ESI) source, whose conditions were as follow: gas temperature, 350 °C, drying gas at 12.0 L/min, nebulizer gas at 35 psig, Vcap at 3000 V, fragmentor at 60V. Unless otherwise indicated, analysis conditions: solvent A = water (with 0.1% HCOOH) and solvent B = acetonitrile (with 0.1% HCOOH). Elution program: flow = 0.5 mL/min; solvent ratio A/B 90/10 during 0.5 min, then a gradient to solvent ratio A/B 10/90 during 5.0 min and then solvent ratio A/B 10/90. Injection volume was typically 0.1 μL. HPLC purities were measured at 254 nm. High Resolution Mass Spectrometry (HRMS) was performed on Bruker microTOF-Q II mass spectrometer.

Experimental procedures for the preparation of compounds **2-6**

**(Piperidin-4-yl)methanol (7)**

A solution of ethyl isonipecotate (77.9 mmol, 12.24 g) in anhydrous THF (80 mL) was added dropwise to an ice-cooled suspension of LiAlH_4_ (97.3 mmol, 3.69 g) in anhydrous THF (250 mL). After 30 min, the mixture was allowed to warm to room temperature and was stirred for 20 h. After addition of water (4.8 mL), 1 M aqueous NaOH (4.8 mL) and water (9.6 mL), the solution was then stirred with diethyl ether (200 mL) for 30 min. The slurry was then filtered and washed with diethyl ether (3 x 100 mL). Solvents were removed under vacuum to afford compound **7** (8.97 g, 100%) as a colorless oil; ^1^H NMR (300 MHz, CDCl_3_): δ = 1.06 – 1.22 (m, 2H), 1.61 (s, 1H), 1.67 – 1.77 (m, 2H), 2.00 (s, 3H), 2.60 (td, *J* = 2.6 Hz and 12.2 Hz, 2H), 3.03 – 3.16 (m, 2H), 3.44 – 3.47 (m, 2H). Spectral data were in accordance to those reported in the literature.^1^

***N*-(2-Chloroethyl)methanesulfonamide**

This compound was prepared according to modified protocols of the literature.^[[5]](#footnote-5),^^[[6]](#footnote-6)^ To a slurry of 2-chloroethylamine hydrochloride (70 mmol, 8.12 g) in MeCN (150 mL) at 5°C was added dropwise (during 30 min) and simultaneously Et_3_N (140 mmol, 19.7 mL) and MeSO_2_Cl (70 mmol, 5.42 mL). After 30 min at 5 °C, the mixture was allowed to warm to RT and the stirring was continued during 3 h. The mixture became slightly orange. After removal of solvent under reduced pressure, the residue was taken off with water (50 mL), the resulting aqueous phase was saturated with solid NaCl and extracted with CH_2_Cl_2_ (6 x 50 mL). The combined organic layers were dried on Na_2_SO_4_, filtered and the solvent was removed under vacuum to give a brown oil (5.87 g) which was purified by flash column chromatography (cyclohexane/EtOAc 6/4) to afford the *title* compound as a yellow oil (3.13 g, 28%) which is solid at -20 °C; ^1^H NMR (300 MHz, DMSO-d_6_): δ = 2.93 (s, 3H), 3.27 (q, *J* = 6.3, 2H), 3.68 (t, *J* = 6.3, 2H), 7.35 (t, *J* = 5.9, 1H). ^13^C NMR (75 MHz, DMSO-d_6_) δ = 39.9, 43.9, 44.3. HRMS (ESI+) m/z: calcd. for C_3_H_8_ClNNaO_2_S: 179.9856; [M+Na]^+^ found: 179.9849.

***N*-(2-Chloroethyl)benzenesulfonamide**

To a mixture of 2-chloroethylamine hydrochloride (43.1 mmol, 5.0 g) solubilized in CH_2_Cl_2_ (100 mL) and water (50 mL) at 10 °C was added K_2_CO_3_ (43.1 mmol, 5.7 g), followed by chlorosulfonylbenzene (43.1 mmol, 5.5 mL). The reaction was vigorously stirred during 3 h, with a temperature kept at 10 °C and the pH value was maintained around 7-8 with K_2_CO_3_. The mixture was then allowed to warm up at RT for 2 h. Layers were partitioned and the aqueous one was extracted with CH_2_Cl_2_ (2 x 100 mL). The volatiles were removed under reduced pressure and, after recrystallization of the crude product in *i*-Pr_2_O (50 mL, 70 °C then RT), the *title* compound was obtained (6.36 g, 67%) as a white solid; mp: 70-72 °C (litt^[[7]](#footnote-7)^: 71-72 °C); ^1^H-NMR (DMSO-d_6_, 300 MHz): δ = 3.08 (q, *J* = 6.1, 2H), 3.57 (t, J = 6.1, 2H), 7.57 – 7.68 (m, 3H), 7.80 – 7.83 (m, 2H), 8.03 (t, *J* = 5.9, 1H). ^13^C NMR (75 MHz, DMSO-d_6_) δ = 43.5, 44.4, 126.4, 129.3, 132.5, 140.4. HRMS (ESI+) m/z: calcd. for C_8_H_10_ClNNaO_2_S: 242.0013; [M+Na]^+^ found: 242.0016.

***N*-[2-[4-(Hydroxymethyl)-1-piperidinyl]ethyl]methylsulfonamide (8a)**

To an ice-cooled solution of (piperidin-4-yl)methanol **7** (67.7 mmol, 7.80 g) in MeCN (70 mL) were added dropwise a solution of *N*-(2-chloroethyl)methanesulfonamide (54.1 mmol, 8.45 g) in MeCN (50 mL). After the addition, the mixture was stirred over 2 days. A 4% aqueous solution of NaOH (200 mL) was then added dropwise. The mixture was extracted with CH_2_Cl_2_ (6 x 100 mL) and the combined organic layers were then dried on MgSO_4_ and filtered. After removal of the solvents under reduced pressure, a yellow oil was obtained, which was further purified by flash column chromatography (CH_2_Cl_2_/absolute EtOH 5/5) to afford the *title* compound as a yellow oil (3.8 g, 30%); ^1^H-NMR (300 MHz, CDCl_3_) δ = 1.20 (t, *J* = 7.0, 2H), 1.51 (s, 1H), 1.69 – 1.77 (m, 2H), 1.97 – 2.06 (m, 2H), 2.45 – 2.57 (m, 2H), 2.87 (d, *J* = 11.6, 2H), 2.96 (s, 3H), 3.10 – 3.25 (m, 2H), 3.49 (d, *J* = 6.3, 2H); ^13^C NMR (75 MHz, DMSO-d_6_) δ = 28.6, 38.3, 39.5, 40.1, 53.2, 57.8, 66,0. HRMS (ESI+) m/z: calcd. for C_9_H_21_N_2_O_3_S: 237.1267; [M+H]^+^ found: 237.1266.

**N-[2-[4-(Hydroxymethyl)-1-piperidinyl]ethyl]phenylsulfonamide (8b)**

To a an ice-cooled solution of piperidin-4-ylmethanol **7** (17.4 mmol, 2.00 g) in MeCN (50 mL) were added diisopropylethylamine (35.0 mmol, 6.1 mL, 4.53 g) and *N*-(2-chloroethyl)benzenesulfonamide (17.4 mmol, 3.82 g). The resulting mixture was heated under reflux for 3 h. The solvent was evaporated under reduced pressure to give a white solid which was washed with water (2 x 75 mL) and dried under vacuum to give the *title* compound as a white solid (3.95 g, 76%); ^1^H NMR (300 MHz, DMSO-d_6_) δ = 1.25 (d, *J* = 14.5, 2H), 1.50 – 1.62 (m, 2H), 1.69 – 1.97 (m, 2H), 2.33 (s, 2H), 2.73 (d, *J* = 9.6, 2H), 2.86 (t, *J* = 6.9, 2H), 3.19 (dd, *J* = 3.8, 6.2, 2H), 4.40 (d, *J* = 6.0, 1H), 7.61 (qdd, *J* = 1.9, 3.6, 8.8, 4H), 7.81 (dd, *J* = 1.7, 8.0, 2H); ^13^C NMR (75 MHz, DMSO-d_6_) δ = 28.6, 38.3, 40.3, 53.1, 57.2, 66.0, 126.5, 129.1, 132.3, 140.6. HRMS (ESI+) m/z: calcd. for C_14_H_23_N_2_O_3_S: 299.1424; [M+H]^+^ found: 299.1424.

***1H*-Indole-3-carboxylic acid (10a)**

Trifluoroacetic anhydride (25 mmol, 3.6 mL) was added dropwise to a solution of *1H*-indole (17.0 mmol, 2.00 g) in *N*,*N*-dimethylformamide DMF (10 mL) at 0 °C. After the end of the addition, the mixture was stirred at room temperature for 3 h. Water (10 mL) was then added and the resulting pink solid was filtered and washed with water. The collected solid was treated with aqueous 20% NaOH solution (40 mL) at 50 °C during 17 h. After cooling to room temperature, the reaction mixture was washed with ether (2 x 30 mL). The aqueous phase was acidified with concentrated HCl (until pH = 2): the desired product crystallised and was collected by filtration. After drying under vacuum, the *title* compound was obtained as a light yellow solid (2.05 g, 75%); ^1^H NMR (300 MHz, DMSO-d_6_) δ = 7.21 (t, *J* = 4.9, 2H), 7.52 (d, *J* = 7.6 Hz, 1H), 7.92 (d, *J* = 7.6 Hz, 1H), 8.14 (s, 1H), 11.71 (s, 1H), 11.81 (s, 1H). Spectral data were in accordance to those reported in the literature.^[[8]](#footnote-8)^

**5-Fluoro-*1H*-indole-3-carboxylic acid (10b)**

Trifluoroacetic anhydride (50 mmol, 7.1 mL) was added dropwise to a solution of 5- fluoro-*1H*-indole (22.2 mmol, 3.00 g) in dry DMF (22 mL) at 0 °C. After the end of the addition, the mixture was heated under reflux for 12 h. Solvent was removed under reduced pressure and the crude product was taken off with water (70 mL) and a solution of NaOH (300 mmol, 12 g) in water (100 mL) was added. The resulting mixture was then heated at reflux for 12 h. After cooling to RT, the solution was washed with Et_2_O (4 x 50 mL) and then acidified with HCl 1 M until pH = 2. The resulting solid was filtered and dried to afford the *title* compound (3.59 g, 90%), as a grey solid; ^1^H NMR (300 MHz, DMSO-d_6_) δ = 7.03 (td, *J* = 2.6, 9.2, 1H), 7.48 (dd, *J* = 4.6, 8.9, 1H), 7.64 (dd, *J* = 2.7, 10.0, 1H), 8.05 (d, *J* = 3.0, 1H), 11.99 (s, 1H). Spectral data are in accordance with literature reference.^[[9]](#footnote-9)^

**(1-(2-(Methylsulfonamido)ethyl)piperidin-4-yl)methyl 5-fluoro-*1H*-indole-3-carboxylate hydrochloride (2)**

*General procedure 1 for the preparation of (1-(2-(methyl-sulfonamido)ethyl)piperidin-4- yl)methyl 1H-indole-3-carboxylate*

Thionyl chloride (5.6 mmol, 406 μL) was slowly added to a suspension of 5-fluoro-*1H*-indole-3- carboxylic acid **10b** (1.12 mmol, 200 mg) in dry CH_2_Cl_2_ (10 mL), and the mixture was the heated under reflux for 24h. After removal of the volatiles under reduced pressure, the residue was immediately put under argon atmosphere then taken off with dry CH_2_Cl_2_ (10 mL) and *N*-[2-[4- (hydroxymethyl)-1-piperidinyl]ethyl]methylsulfonamide **8a** (1.10 mmol, 260 mg) was added. The resulting mixture was heated under reflux for 24h. After removal of the solvent under vacuum, the resulting brown solid was dissolved in water (50 mL). The aqueous layer was then saturated with solid NaCl and extracted with CH_2_Cl_2_ (5 x 50 mL). The combined organic layers were washed with brine, dried on Na_2_SO_4_, filtered and solvents were removed under vacuum. The resulting grey solid was purified by flash column chromatography (CH_2_Cl_2_/MeOH 90/10 to 75/25) to afford a brown foam (134 mg, 30 %), which was directly engaged in the hydrochloride salt formation step.

*General procedure 2 for the preparation of hydrochloride salts:*

To a solution of the previous compound (1-(2-(methylsulfonamido)ethyl)piperidin-4-yl)methyl 5-fluoro-*1H*-indole-3-carboxylate (0.16 mmol, 62 mg) in MeOH (3 mL) was slowly added acetyl chloride (0.48 mmol, 34 μL). After removal of solvent under reduced pressure, the residue was taken off with CHCl_3_ (5 mL) and the volatiles were evaporated. This operation was repeated two other times. The same protocol was done with Et_2_O (3 x 5 mL) to evaporate all the remaining volatiles and to afford the *title* compound as a yellow solid (73 mg, quantitative); ^1^H NMR (400 MHz, DMSO-d6) δ = 12.24 (s, 1H), 10.61 (s, 1H), 8.27 – 8.18 (m, 1H), 7.62 (dd, *J* = 2.8, 9.8, 1H), 7.51 (dd, *J* = 4.6, 9.1, 2H), 7.06 (td, *J* = 2.7, 9.2, 1H), 4.23 – 4.07 (m, 2H), 3.53 (s, 3H), 3.15 (s, 2H), 2.98 (s, 3H), 2.11 – 1.84 (m, 4H), 1.76 (d, *J* = 13.1, 2H), 1.35 (d, *J* = 5.8, 2H). ^13^C NMR (101 MHz, DMSO-d6) δ = 25.7, 26.7, 30.4, 33.0, 34.4, 37.2, 51.7, 55.7, 66.2, 105.2, 106.4, 110.8, 113.8, 126.3, 133.1, 157.1, 159.5, 164.0; LC-MS (ESI+): 96 % pure at 254 nm, *m/z* [M + H]^+^: 398.2. HRMS (ESI) *m/z*: calcd. for C_18_H_25_FN_3_O_4_S: 398.1550; [M+H]^+^ found: 398.1536.

**(1-(2-(Methylsulfonamido)ethyl)piperidin-4-yl)methyl *1H*-indole-3-carboxylate (3)**

The reaction was carried out according to the general procedure 1, scale: *1H*-indole-3-carboxylic acid **10a** (1.67 mmol, 270 mg), SOCl_2_ (8.35 mmol, 606 μL), CH_2_Cl_2_ (10 mL), then *N*-[2-[4-(hydroxymethyl)-1-piperidinyl]ethyl]methylsulfonamide **8a** (1.58 mmol, 373 mg), CH_2_Cl_2_ (10 mL). The crude was directly purified by flash column chromatography (CH_2_Cl_2_/MeOH 90/10) to afford the *title* compound as a brown foam (313 mg, 50 %). ^1^H NMR (400 MHz, DMSO-d6) δ = 1.75 (s, 2H), 1.83 – 1.94 (m, 2H), 1.93 – 2.05 (m, 1H), 2.92 (s, 2H), 2.97 (s, 3H), 3.07 (q, *J* = 7.7, 8.1, 2H), 4.14 (d, *J* = 5.9, 2H), 7.14 – 7.26 (m, 2H), 7.41 – 7.54 (m, 2H), 7.96 – 8.02 (m, 1H), 8.15 (d, *J* = 3.0, 1H), 10.81 (s, 1H), 12.14 (d, *J* = 3.1, 1H). ^13^C NMR (101 MHz, DMSO-d6) δ = 18.6, 33.2, 37.6, 38.9, 40.1, 40.2, 51.8, 56.0, 66.2, 106.2, 107.0, 112.4, 116.1, 120.4, 121.4, 122.4, 125.6, 132.8, 136.5, 164.4; LC-MS (ESI+): 98 % pure, *m/z* [M + H]^+^: 380.2, HRMS (ESI+) *m/z*: calcd. for C_18_H_26_N_3_O_4_S: 380.1639; [M+H]^+^ found: 380.1629.

**(1-(2-(Phenylsulfonamido)ethyl)piperidin-4-yl)methyl *1H*-indole-3-carboxylate (4)**

*General procedure 3 for the preparation of (1-(2-(phenylsulfonamido)ethyl)piperidin-4-yl)methyl 1H-indole-3-carboxylate.*

Five drops of DMF were added to a solution of 1*H*-indole-3-carboxylic acid **10a** (11.8 mmol, 1.90 g) in dry CH_2_Cl_2_ (5 mL), followed by a slow addition of a solution of thionyl chloride (17.7 mmol, 1.28 mL) in dry CH_2_Cl_2_ (5 mL). The resulting mixture was stirred for 48h at room temperature, until the complete consumption of the carboxylic acid, monitored by LC-MS analysis. The pale yellow solution was concentrated under reduced pressure to give a pale beige solid (1.95 g, 92%), that was used without further purification. ^1^H NMR (DMSO-d_6_, 400 MHz): δ = 7.36 (2H, m, ArH), 7.54 (1H, m, ArH), 8.13 (1H, d, J = 3.5 Hz, ArH), 8.34 (1H, s, ArH), 9.1 (1H, br, NH). Spectral data are in agreement with literature data.^[[10]](#footnote-10)^

This pale beige solid (10 mmol, 1.80 g) was dissolved in dry CH_2_Cl_2_ (10 mL) and *N*-[2-[4- (hydroxymethyl)-1-piperidinyl]ethyl]phenylsulfonamide **8b** (10,2 mmol, 3,05 g) was added. The resulting mixture was vigorously heated at reflux for 24 h. After cooling to room temperature and removal the solvent, the resulting oil was purified flash chromatography (CH_2_Cl_2_/MeOH/25% aqueous NH_3_ 95.5/4/0.5) to give the *title* compound as a white foam (3.35 g, 76%); m.p. 54-57 °C. ^1^H NMR (300 MHz, DMSO-d_6_) δ = 1.24 (d, J = 9.1, 2H), 1.66 (d, J = 11.4, 3H), 1.87 (t, J = 11.1, 2H), 2.28 (t, J = 6.8, 2H), 2.72 (d, J = 10.6, 2H), 2.85 (t, J = 6.9, 2H), 4.07 (d, J = 5.9, 2H), 7.15 – 7.22 (m, 2H), 7.45 – 7.50 (m, 1H), 7.52 – 7.68 (m, 3H), 7.77 – 7.85 (m, 2H), 7.94 – 8.00 (m, 1H), 8.07 (d, J = 2.7, 1H), 11.94 (s, 1H). ^13^C NMR (101 MHz, DMSO-d_6_) δ = 28.5, 35.2, 40.3, 52.8, 57.1, 67.2, 106.5, 112.4, 120.4, 121.3, 122.3, 125.6, 126.5,129.14, 132.3, 132.5, 136.4, 140.6, 164.4. LC-MS (ESI+): 93% pure at 254 nm, m/z [M + H]^+^: 442.2. HRMS (ESI+) *m/z*: calcd. for C_23_H_28_N_3_O_4_S: 442.1795; [M+H]^+^ found: 442.1786.

**(1-(2-(Phenylsulfonamido)ethyl)piperidin-4-yl)methyl 5-fluoro-1*H*-indole-3-carboxylate hydrochloride (5)**

The reaction was carried out according to general procedure 3, scale: 5-fluoro-1*H*-indole-3-carboxylic acid **10b** (9.0 mmol, 1.78 g), DMF (5 drops), SOCl_2_ (mmol, 1.73 mL,), CH_2_Cl_2_ (10 mL). Second step: *N*-[2-[4-(hydroxymethyl)-1-piperidinyl]ethyl]phenylsulfonamide **8b** (9.2 mmol, 2.74 g), CH_2_Cl_2_ (10 mL), 24 h heating at reflux. The crude product was purified by flash chromatography (CH_2_Cl_2_/MeOH/aqueous NH_3_ 95.5/4/0.5) to afford the product as a white foam (2.60 g, 63%). LC-MS (ESI+): m/z [M + H]^+^: 460.2 The product was directly engaged in the hydrochloride salt formation next step.

The hydrochloride salt formation was carried out according to general procedure 2, scale: (1-(2-(phenylsulfonamido)ethyl)piperidin-4-yl)methyl 5-fluoro-1*H*-indole-3-carboxylate (0.28 mmol, 131 mg), MeOH (3 mL), acetyl chloride (0.86 mmol, 61 μL). After removal of solvent under reduced pressure, the residue was washed with diethyl ether (3 x 3 mL). MeOH (3 mL) was added to the resulting solid (120 mg) and heated at reflux for 15 min. The insoluble part was separated and washed with diethyl ether (2 x 3 mL). This operation was repeated two times to obtain the *title* compound as a white solid (64 mg, 46%); m.p.: 227-228 °C. ^1^H NMR (400 MHz, DMSO-d_6_) δ = 1.74 (tt, J = 7.1, 13.6, 2H), 1.89 (d, J = 13.6, 2H), 1.93 – 2.07 (m, 1H), 2.97 (q, J = 11.3, 2H), 3.07 – 3.27 (m, 5H), 3.40 – 3.54 (m, 2H), 4.13 (d, J = 5.9, 2H), 7.06 (td, J = 2.7, 9.2, 1H), 7.50 (dd, J = 4.6, 8.9, 1H), 7.58 – 7.72 (m, 4H), 7.85 (dd, J = 1.8, 7.0, 2H), 8.18 (d, J = 5.6, 1H), 8.23 (d, J = 3.1, 1H), 10.53 (s, 1H), 12.24 (d, J = 3.4, 1H). ^13^C NMR (100 MHz, DMSO-d_6_) δ = 25.5, 32.8, 37.1, 40.2, 48.6, 51.6, 55.3, 66.1, 105.0, 105.2, 106.3, 106.4, 110.6, 110.8, 113.7, 113.8, 126.2, 126.3, 126.6, 126.6, 129.4, 132.8, 133.1, 134.4, 139.7, 157.1, 159.4, 164.0. LC-MS (ESI+): 98 % pure at 254 nm, m/z [M + H]^+^: 460.2. HRMS (ESI) m/z: calcd. for C_23_H_29_FN_3_O_4_S: 460.1701 [M+H]^+^; found: 460.1704.

**1-(2-(Phenylsulfonamido)ethyl)piperidin-4-yl)methyl 5-fluoro-2-methoxy-1*H*-indole-3-carboxylate hydrochloride (6)**

To a solution of (1-(2-(phenylsulfonamido)ethyl)piperidin-4-yl)methyl 5-fluoro-1*H*-indole-3-carboxylate (0.32 mmol, 148 mg) in dry CHCl_3_ (7 mL) was added *N*-chlorosuccinimide (0.39 mmol, 52 mg). The mixture was heated at reflux under argon for 4 h. After removal of solvent under reduced pressure, the residue was taken off with MeOH (10 mL) and heated at reflux under argon for 12 h. After removal of solvent under vacuum, the resulting brown foam (207 mg) was purified by flash chromatography (CH_2_Cl_2_/MeOH 9/1) to afford a brown foam (0.22 mmol, 109 mg). Yield = 56%. LC-MS (ESI+): 99% pure at 254 nm, m/z [M + H]^+^: 490.2. The product was directly engaged in the next step.

The hydrochloride salt formation was carried out according to the general procedure 2, scale: (1-(2-(phenylsulfonamido)ethyl)piperidin-4-yl)methyl 5-fluoro-2-methoxy-1*H*-indole-3-carboxylate (0.22 mmol, 109 mg), MeOH (3 mL), acetyl chloride (0.70 mmol, 50 μL). The crude oil was dissolved in boiling methanol (3 mL) and then cooled to -18 °C overnight. A white precipitate occurred. After removal of MeOH, the resulting solid was washed with methanol (1 x 2 mL) and with diethyl ether (3 x 2 mL). MeOH (3 mL) was added to the resulting solid and heated at reflux for 15 min. The insoluble part was separated and then washed with diethyl ether (2 x 3 mL). This purification was repeated a second time to give the *title* compound as a white solid (38 mg, 33 %); mp: 195-196°C; ^1^H-NMR (400 MHz, DMSO-d_6_) δ = 1.54 – 1.69 (m, 2H), 1.88 (d, J = 12.9, 2H), 1.97 (s, 1H), 2.96 (q, J = 11.2, 2H), 3.11 (t, J = 5.6, 2H), 3.16 (s, 1H), 3.18 (d, J = 6.3, 2H), 3.40 (s, 2H), 3.48 (d, J = 12.0, 2H), 4.07 (d, J = 6.4, 2H), 4.11 (s, 3H), 6.88 (ddd, J = 2.7, 8.6, 9.6, 1H), 7.30 (dd, J = 4.7, 8.7, 1H), 7.45 (dd, J = 2.7, 10.2, 1H), 7.59 – 7.71 (m, 3H), 7.82 – 7.88 (m, 2H), 8.16 (t, J = 5.9, 1H), 10.42 (d, J = 67.8, 1H), 12.27 (s, 1H). ^13^C NMR (101 MHz, DMSO-d_6_) δ = 25.8, 33.0, 37.1, 38.9, 39.1, 39.3, 39.5, 39.6, 39.7, 39. 8, 39.9, 40.0, 40.2, 40.2, 48.6, 51.6, 55.4, 58.8, 65.6, 86.5, 86.5, 104.6, 104.9, 107.8, 108.1, 112.1, 112.2, 126.6, 126.9, 127.1, 127.2, 129.4, 132.8, 139.7, 157.2, 158.5, 159.5, 163.2. LC-MS (ESI+): 99% pure at 254 nm, m/z [M + H]^+^: 490.2. HRMS (ESI+) m/z: calcd. for C_24_H_30_FClN_3_O_5_S: 490.1806; [M+H]^+^ found: 498.1800.

1. Boyer, N., Gloanec, P., De Nanteuil, G., Jubault, P. & Quirion, J.-C. Synthesis of α,α-difluoro-β-amino esters or gem-difluoro-β-lactams as potential metallocarboxypeptidase inhibitors. *Eur. J. Org. Chem.* 4277-4295 (2008). [↑](#footnote-ref-1)
2. Wang, X., Liu, Y., Xu, J., Jiang, F. & Kang, C. Synthesis of novel indole-benzimidazole derivatives. *J. Chem. Res.* **40**, 588-590 (2016). [↑](#footnote-ref-2)
3. Oxford, A. W., Whitehead, J. W. F. & Knight, J. *Eur. Patent Appl.* 0501322 A1 (1992). [↑](#footnote-ref-3)
4. Gottlieb, H. E.; Kotlyar, V.; Nudelman, A. NMR chemical shifts of common laboratory solvents as trace impurities. *J. Org. Chem.* **62**, 7512-7515 (1997). [↑](#footnote-ref-4)
5. Suda, H., Kanda, T., Tomita, H., Nakanishi, H., Hida, H., Nuno, T., Akutsu, S. & Maeyashiki, M. Process for producing sulfonylamides. *U.S. Patent Appl.* 3,956,385 (1976). [↑](#footnote-ref-5)
6. Lee, J., Zhong, Y.-L., Reamer, R. A. & Askin, D. Practical synthesis of sultams via sulfonamide dianion alkylation: application to the synthesis of chiral sultams. *Org. Lett.* **5**, 4175–4177 (2003). [↑](#footnote-ref-6)
7. Coy, J. H., Hegarty, A. F., Flynn, E. J. & Scott, F. L. Ambident Neighbouring Groups. Part V. Mechanism of Cyclization of 2-Halogenoethylsulphonamides to Aziridines. *J. Chem. Soc., Perkin Trans. 2* 53-58 (1974). [↑](#footnote-ref-7)
8. Nemoto, K., Tanaka, S., Konno, M., Onozawa, S., Chiba, M., Tanaka, Y., Sasaki, Y., Okubo, R. & Hattori, T. Me_2_AlCl-mediated carboxylation, ethoxycarbonylation, and carbamoylation of indoles. *Tetrahedron* **72** 734-745 (2016). [↑](#footnote-ref-8)
9. Yoo, W.-J., Guiteras Capdevila, M., Du, X. & Kobayashi, S. *Org. Lett.* **14**, 5326-5329 (2012). [↑](#footnote-ref-9)
10. Abuhaie, C.-M., Bîcu, E., Rigo, B., Gautret, P., Belei, D., Farce, A., Dubois, J. & Ghinet, A. Bioorg. Med. Chem. Lett. 23, 147 – 152 (2013). [↑](#footnote-ref-10)
